# Actionable Mutation Profiles of Non-Small Cell Lung Cancer patients from Vietnamese population

**DOI:** 10.1038/s41598-020-59744-3

**Published:** 2020-02-17

**Authors:** Anh-Thu Huynh Dang, Vu-Uyen Tran, Thanh-Truong Tran, Hong-Anh Thi Pham, Dinh-Thong Le, Lam Nguyen, Ngoc-Vu Nguyen, Thai-Hoa Thi Nguyen, Chu Van Nguyen, Ha Thu Le, Mai-Lan Thi Nguyen, Vu Thuong Le, Phuc Huu Nguyen, Binh Thanh Vo, Hong-Thuy Thi Dao, Luan Thanh Nguyen, Thien-Chi Van Nguyen, Quynh-Tram Nguyen Bui, Long Hung Nguyen, Nguyen Huu Nguyen, Quynh-Tho Thi Nguyen, Truong Xuan Le, Thanh-Thuy Thi Do, Kiet Truong Dinh, Han Ngoc Do, Minh-Duy Phan, Hoai-Nghia Nguyen, Le Son Tran, Hoa Giang

**Affiliations:** 1 University of Medicine and Pharmacy at Ho Chi Minh city, Ho Chi Minh city, Vietnam; 2 Gene Solutions, Ho Chi Minh city, Vietnam; 3 Pham Ngoc Thach Hospital, Ho Chi Minh city, Vietnam; 4 Vietnam National Cancer Hospital, Ha Noi, Vietnam; 5 Ha Noi Oncology hospital, Ha Noi, Vietnam; 6 Cho Ray hospital, Ho Chi Minh City, Vietnam; 7 Medical Genetics Institute, Ho Chi Minh City, Vietnam; 8 School of Chemistry and Molecular Biosciences, University of Queensland, Brisbane, Australia; 9 Present Address: Institute of Molecular and Cellular Biology, Astar, Singapore

**Keywords:** Non-small-cell lung cancer, Genetic testing

## Abstract

Comprehensive profiling of actionable mutations in non-small cell lung cancer (NSCLC) is vital to guide targeted therapy, thereby improving the survival rate of patients. Despite the high incidence and mortality rate of NSCLC in Vietnam, the actionable mutation profiles of Vietnamese patients have not been thoroughly examined. Here, we employed massively parallel sequencing to identify alterations in major driver genes (*EGFR*, *KRAS, NRAS, BRAF*, *ALK* and *ROS1*) in 350 Vietnamese NSCLC patients. We showed that the Vietnamese NSCLC patients exhibited mutations most frequently in *EGFR* (35.4%) and *KRAS* (22.6%), followed by *ALK* (6.6%), *ROS1* (3.1%), *BRAF* (2.3%) and *NRAS* (0.6%). Interestingly, the cohort of Vietnamese patients with advanced adenocarcinoma had higher prevalence of *EGFR* mutations than the Caucasian MSK-IMPACT cohort. Compared to the East Asian cohort, it had lower *EGFR* but higher *KRAS* mutation prevalence. We found that *KRAS* mutations were more commonly detected in male patients while *EGFR* mutations was more frequently found in female. Moreover, younger patients (<61 years) had higher genetic rearrangements in *ALK* or *ROS1*. In conclusions, our study revealed mutation profiles of 6 driver genes in the largest cohort of NSCLC patients in Vietnam to date, highlighting significant differences in mutation prevalence to other cohorts.

## Introduction

Lung cancer is the most common malignancy and the leading cause of cancer related deaths worldwide (18.4% of total cancer deaths), with non-small cell lung cancer (NSCLC) being the most common subtype, accounting for approximately 85% of all diagnosed cases^1,2^. The majority of NSCLC patients display advanced disease when diagnosed and thus have poor prognosis^2,3^. It is well established that acquired genetic alterations in certain driver genes result in tumour growth and invasiveness, and that patients harboring certain mutations may benefit from targeted therapies^4,5^. Indeed, a randomized clinical trial reported that advanced NSCLC patients harboring activating mutations in *EGFR*, one of the major driver genes of NSCLC, exhibited longer progression-free period when treated with a tyrosine kinase inhibitor (TKI), gefitinib, compared to those treated with standard platinum based chemotherapy^6^. However, those who were treated with TKI drugs can acquire secondary resistant mutations, in which case a new treatment regimen is needed to maintain therapeutic effect^7,8^. In addition to *EGFR*, NSCLC patients carrying *ALK* or *ROS1* rearrangement were shown to respond well to a different TKI drug, crizotinib, while *BRAF* mutated NSCLC patients can be treated with a combination of BRAF inhibitors, dabrafenib and trametinib^9–12^. These findings suggest that the identification of mutation profiles of NSCLC is critical in order to prescribe suitable TKI therapy as well as elucidate the molecular basis of drug resistance to provide timely treatment adjustment.

Since 2018, the American Society of Clinical Oncology (ASCO) has recommended routine mutation testing for driver genes including *EGFR*, *ALK*, *ROS1* and *BRAF* in clinical practice for NSCLC patients^13^. Although there are currently no targeted drugs for *KRAS* or *NRAS* mutated NSCLCs, mutation testing for these genes has also been recommended due to their proven impact on clinical outcomes of NSCLC patients^14,15^. Hence, simultaneous mutation profiling of these six driver genes has been recommended in current clinical practice^13,16,17^. Currently, information in publicly available database such as The Cancer Genome Atlas (TCGA) was obtained mostly from prospective studies in Caucasians and European cohorts. Therefore, the impact of heterogeneous genetic constitution of NSCLC patients across different populations might be underestimated^17^.

Vietnam displays higher incidence rate of lung cancer than the average worldwide incidence (14.5% versus 11% of all new cases) despite having the same level of mortality rate according to the latest Globocan data^18,19^. Also, in Vietnam lung cancer is the most aggressive type of cancer in men and ranks as the second leading cause of cancer deaths in women^18,19^. Therefore, it is important to assess the mutation landscape as well as their association with unique clinicopathological features of Vietnamese NSCLC patients. In the present study, we employed massively parallel sequencing to detect genetic changes in six major driver genes including *EGFR*, *KRAS*, *NRAS*, *BRAF*, *ALK* and *ROS1* in tumour tissues from 350 NSCLC patients in Vietnam. Interestingly, we found that the Vietnamese cohort had a significantly higher frequency of *KRAS* mutations as compared to Caucasians and East Asian cohorts. We further identified significant associations between the prevalence of these mutations with patients’ clinical features in the Vietnamese cohort. Our data provide valuable information for guiding targeted therapy and drug development for Vietnamese NSCLC patients.

## Results

### Clinical characteristics of Vietnamese NSCLC patients

The cohort in this study comprised of 350 patients diagnosed with NSCLC by clinical histology from 4 hospitals in Vietnam, with higher percentage of male compared to female (60.6% versus 37.4%, p < 0.05) and the median age of 61 years, ranging from 24 to 89 years (Table 1). The majority of patients (289 cases, 82.6%) were classified into advanced stages (III-IV), while 12 patients (3.4%) were in early stages (I-II) and 49 cases (14%) missing information on clinical stages (Table 1). Adenocarcinoma (AC) was the most common NSCLC subtype (241 cases, 68.9%) while squamous carcinoma (SCC) was confirmed in 25 patients (7.1%). Additionally, 84 cases (24%) were of either unknown or uncharacterized subtypes (Table 1). Among 350 patients, 185 cases (52.8%) in this cohort had smoking status recorded including 54 smokers (15.4%) and 131 non-smokers (37.4%) while 165 cases (47.1%) had missing smoking status (Table 1). Treatment details were obtained for 306 patients (87.4%), including 285 patients (81.4%) who had never taken any treatment at the time of diagnosis and 21 patients (6%) experienced either tumour resection (5 cases, 1.4%), chemotherapy combined with radiotherapy (10 cases, 2.9%) or TKI therapy (6 cases, 1.7% for each treatment) (Table 1).

**Table 1:** Major clinicopathological features of 350 Vietnamese NSCLC patients N: number of cases; AC: adenocarcinoma; SCC: squamous cell carcinoma.

Clinical characteristics		N	%
Sex	Female	131	37.4
	Male	212	60.6
	Unknown	7	2
Age	< = 61	174	49.7
	>61	170	48.6
	Unknown	6	1.7
Smoking status	Yes	54	15.4
	No	131	37.4
	Unknown	165	47.1
Histology	AC	241	68.9
	SCC	25	7.1
	Others or unknown	84	24
Tumour Location	Lung	231	66
	Bronchi	66	18.9
	Others or unknown	53	15.1
Tumour Stage	I-II	12	3.4
	III-IV	289	82.6
	Unknown	49	14.0
Treatment information	Naïve to treatment	285	81.4
	Resection	5	1.4
	Chemotherapy /Radiation	10	2.9
	TKI	6	1.7
	Unknown	44	12.6

### Mutation profiles of driver genes in Vietnamese NSCLC patients

In the present study, we developed a targeted capture sequencing assay to analyse genetic alterations in formalin-fixed paraffin-embedded (FFPE) tissue biopsy specimens of NSCLC patients. We first validated our assay by comparing its performance with a commercial droplet digital PCR (ddPCR) assay (Bio-rad) for detecting three major *EGFR* mutations (L858R, del19 and T790M) in 40 tissue samples randomly selected from our cohort. When ddPCR results were used as reference standard, our targeted capture sequencing assay exhibited sensitivity of 81.8% (11/13), specificity of 100% (27/27) and concordance rate of 95% (38/40) (Table 2, Table S2). The two cases (LBL021 and L10021) that were positive for del19 mutation by ddPCR but missed by our assay had relatively low variant allele frequency (VAF) of 0.5% and 3.9%, respectively, below the limit of detection of our assay (4%) (Table S2). Hence, these results confirmed that our targeted capture sequencing assay achieved precise identification of mutations with VAF >4% in FFPE tissue samples. Therefore, this assay was subsequently used to identify genetic alterations in six major driver genes of NSCLC including *KRAS*, *EGFR*, *NRAS*, *BRAF*, *ALK* and *ROS1* for the cohort of 350 NSCLC patients.

**Table 2:** Comparison of *EGFR* mutation detection between our targeted capture sequencing assay and a commercial ddPCR assay.

ddPCR	NGS			Results	
	Mutation	Wild type	Total		
Mutation	11	2	13	Sensitivity (%)	84.6% (95% CI:54.5%–98.1%)
Wild type	0	27	27	Specificity (%)	100% (95% CI:87.2%–100%)
Total	11	29	40	Concordance (%)	95% (95% CI:83.1%–99.4%)

**Figure 1:**
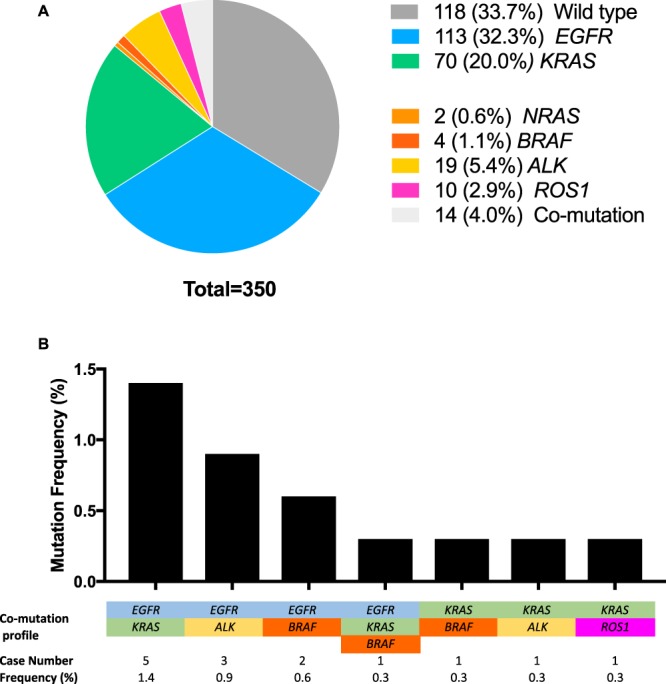
The mutation composition in six major driver genes in 350 Vietnamese NSCLC patients. (**A**) Prevalence of mutations in 6 major driver genes determined by targeted capture sequencing. Cases with mutations occurring in more than one driver gene were counted as “co-mutation”. (**B**) Mutation frequency in cases harbouring co-mutations.

Among 350 patients successfully sequenced, 232 (66.3%) cases were found to carry at least one clinically relevant genetic alteration (according to ClinVar) in the tested driver genes while the remaining 118 cases (33.7%) were negative for these mutations (Fig. 1A). *EGFR* (32.3%) and *KRAS* (20%) were the most frequently mutated driver genes, followed by *ALK* (5.4%), *ROS1* (2.9%), *BRAF* (1.1%) and *NRAS* (0.6%) **(**Fig. 1A**)**. Although mutations in driver genes such as *EGFR*, *KRAS* and *ALK* were reported to be mutually exclusive in majority of NSCLC patients^20^, we detected 14 cases (4%) carrying mutations in more than one driver genes **(**Fig. 1B**)**. Of those, the co-occurrence of mutations in *EGFR* and *KRAS* was the most common (6 cases), including one case carrying concurrent mutations in 3 driver genes (*EGFR*, *KRAS* and *BRAF*). *EGFR* mutation was also found in 3 cases with *ALK* rearrangement and 2 cases with *BRAF* mutation. In addition, *KRAS* mutations were also detected in patients carrying *BRAF* (2 cases), *ALK* (1 case) and *ROS1* (1 case) mutations (Fig. 1B).

**Figure 2:**
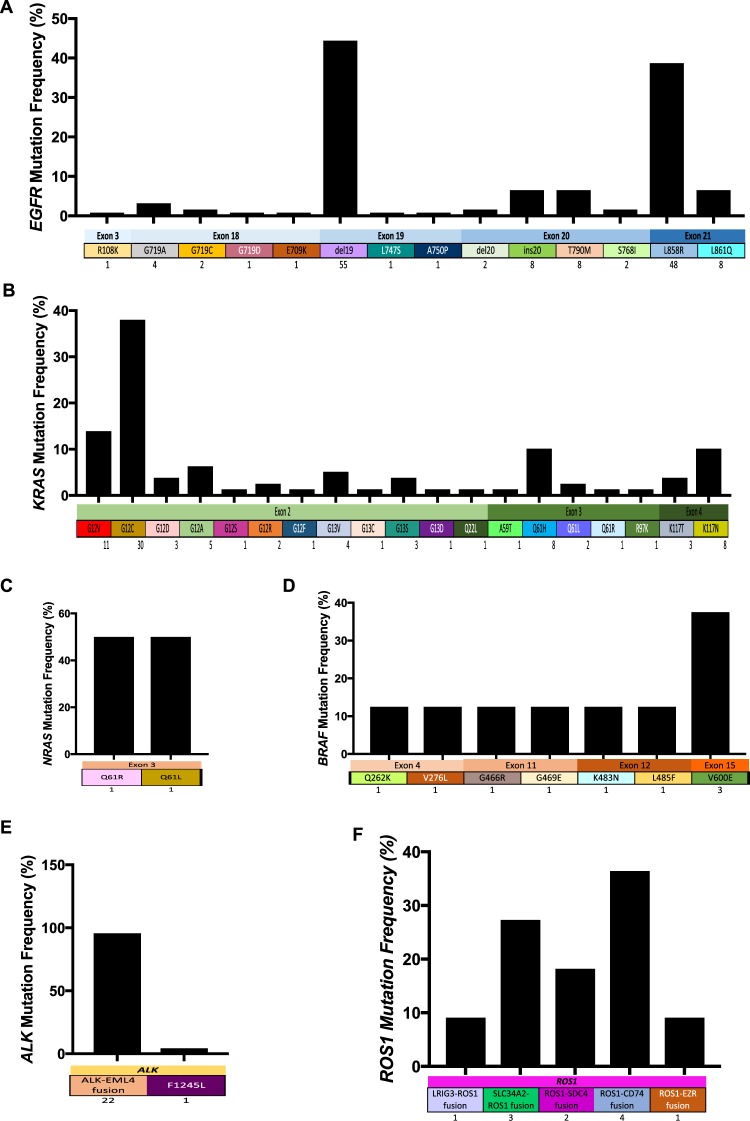
Distribution of mutation subtypes across 6 driver genes in Vietnamese NSCLC. (**A–F**) The mutation frequencies in particular subtypes of *EGFR* (**A**), *KRAS* (**B**), *BRAF* (**C**), *NRAS* (**D**), *ALK* (**E**) and *ROS1* (**F**) genes were calculated as percentage of mutant cases in the total number of cases carrying mutations in the corresponding driver gene.

We next explored the distribution of mutation sites and subtypes across the six driver genes. *EGFR* mutations were predominantly detected in exon 19 and exon 21, with activating mutations del19 (55 cases) and L858R (48 cases) accounting for the majority of *EGFR* mutations (83.1% of all *EGFR* mutation events) (Fig. 2A). In contrast, mutations reported to be associated with resistance to TKI therapy, including T790M and ins20 in exon 20, were detected in 16 patients (12.9% of total patients, 8 cases for each mutation type) (Fig. 2A). All ins20 mutations were later confirmed by a real time-PCR based commercial assay Cobas^®^ *EGFR* Mutation Test (Roche) (Data not shown). Additionally, other rare mutations with prevalence of less than 1% were also detected in exon 3 (R108K) and exon 18 (G719A, G719C, G719D and E709K) (Fig. 2A). The most common *KRAS* mutations occurred in exon 2 at G12 and G13 residues, accounting for 62/79 cases (78.4% of total *KRAS* mutation cases, Fig. 2B). Mutations were also detected in exon 3, residues Q61 (11 cases, 13.9%) and exon 4, residue K117 (11 cases, 13.9%, Fig. 2B). There were 8 cases carrying more than one *KRAS* mutation subtype and such concurrent mutations tend to arise at the same residue, with 6 out of 8 cases carrying co-mutations at the same residue (1 case at G12, 4 cases at G13 and 1 case at Q61) (Fig. 2B). Mutations in *NRAS* were detected in exon 3, residue Q61 (Fig. 2C); and mutations in *BRAF* were detected in exon 4, 11 and 12 (2 cases each) and exon 15, residue V600 (3 cases) (Fig. 2D).

Our assay was able to detect genetic rearrangements in *ALK* and *ROS1*. ALK-EML4 translocation was identified as the most common mutation subtype (22/23 cases, 95.6%, Fig. 2E). All *ROS1* mutations (11 cases) identified in this cohort were the results of fusion with the following genes: *LIRG3* (1 case), *SLC34A2* (3 cases), *SDC4* (2 cases), *CD74* (4 cases) and *ERZ* (1 case) (Fig. 2F).

Collectively, our data indicated that mutations in *EGFR*, *KRAS* are the two most common events occurring in more than half of all tested NSCLC patients in Vietnam, followed by less frequent mutations in other tested genes.

### Comparison of mutation profiles among three NSCLC cohorts

To put the mutation profiles found in the Vietnamese cohort into global context, we compared the prevalence of mutations in Vietnamese NSCLC patients with those found in two other cohorts including the Caucasian MSK-IMPACT cohort established by the Memorial Sloan Kettering (MSK) Cancer Center^21,22^ and East Asian (China) cohort retrieved from a study by Wang *et al*.^23^ (Table 3). Since the majority of patients in the Vietnamese cohort were diagnosed with AC subtype in advanced stages (III-IV), we selectively retrieved data from patients with comparable histology and tumour stage from the other two cohorts. Given the comparable histology and stage, the Vietnamese cohort had fewer female patients than the other two cohorts. Additionally, patients in this cohort were slightly younger than the MSK-IMPACT cohort (median age: 61 versus 64, p < 0.001, Table 3) but older than the East Asian cohort (median age: 61 versus 58, p < 0.001, Table 3).

**Table 3:** Comparison of mutation frequency among the three cohorts.

Characteristics		Vietnam (N = 220)		MSK-IMPACT (N = 764)			East Asia (N = 361)		
		N	%	N	%	p	N	%	p
Sex	Female	90	40.9	447	58.5	<0.00001	201	55.7	<0.001
	Male	128	58.2	317	41.5		160	44.3	
	UN	2	0.9	0	0.0		0	0	
Age at diagnosis	Min	31		19		<0.001	22		<0.001
	Max	86		93			84		
	Median	61		64			58		
*EGFR*	Mutant	83	37.7	222	29.1	<0.05	265	73.4	<0.00001
	WT	137	62.3	542	70.9		96	26.6	
*KRAS*	Mutant	47	21.4	188	24.6	NS	33	9.1	<0.0001
	WT	173	78.6	576	75.4		328	90.9	
*NRAS*	Mutant	1	0.5	10	1.3	NS	Not tested		
	WT	219	99.5	754	98.7				
*BRAF*	Mutant	5	2.3	26	3.4	NS	5	1.4	NS
	WT	215	97.7	738	96.6		356	98.6	
*ALK*	Mutant	17	7.7	30	3.9	NS	31	10.0	NS
	WT	203	92.3	734	96.1		330	90.0	
*ROS1*	Mutant	5	2.3	23	3.0	NS	6	1.7	NS
	WT	215	97.7	741	97.0		355	98.3	

N: total case number

n: number of cases

%: percentage of cages

UN: unknown

WT: wild type

Kruskal-Wallis and Dunn’s multiple comparison test was conducted to compare the median age of patients at diagnosis

Chi-squared (χ²) test was performed to compare gender and mutation frequencies and the p-values were subsequently adjusted by Bonferroni correction.

*NRAS* mutations were not tested in the East Asian cohort.

**Figure 3:**
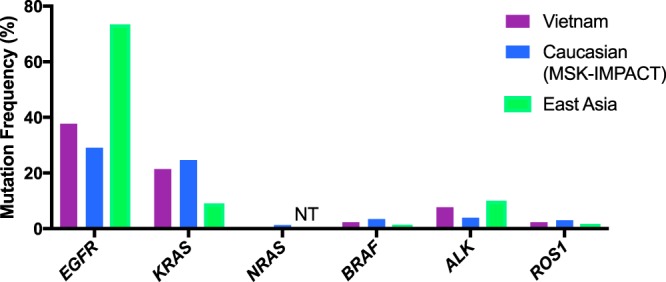
Comparison of driver gene mutation frequencies between Vietnamese NSCLC cohort with Caucasians and East Asians. Mutation frequency of each driver gene in the Vietnamese cohort was calculated among 220 patients with adenocarcinoma (AC) in late stages (III-IV) taking into account cases with co-mutation. For the Caucasian cohort, data were obtained from the MSK-IMPACT cohort (764 lung cancer cases with AC subtypes in metastatic stages (III-IV), Asian patients were excluded). For East Asia cohort, data we retrieved from a recent report profiling a similar panel of driver mutations in a Chinese cohort of 361 patients with AC in late stages (III-IV). NT: mutations were not tested.

The prevalence of mutations in *EGFR* was significantly higher in the Vietnamese cohort compared to the MSK-IMPACT cohort (37.7% versus 29.1%, p < 0.05, Table 3 and Fig. 3) but markedly lower than the East Asian cohort (37.7% versus 73.4%, p < 0.00001, Table 3 and Fig. 3). Interestingly, the prevalence of *KRAS* mutations of the Vietnamese cohort was comparable to the MSK-IMPACT cohort while it was significantly higher than that of East Asians (21.4% versus 9.1%, p < 0.0001, Table 3, Fig. 3**)**. Apart from *KRAS* and *EGFR* mutations, mutation frequencies of the remaining tested genes showed no significant differences between the Vietnamese cohort and the other two cohorts (Table 3, Fig. 3**)**

In summary, the cohort of Vietnamese NSCLC patients showed specific characteristics that set it apart from the two other cohorts. It had higher prevalence of *EGFR* mutations than the Caucasian MSK-IMPACT cohort but lower than the East Asian cohort while its *KRAS* mutation prevalence is higher than the East Asian cohort.

### Correlation between mutation prevalence and clinicopathological features of Vietnamese NSCLC patients

Previous studies have reported significant association between prevalence of driver mutations and patients’ clinicopathological features^24–29^. However, the results are often inconsistent across different studies. Here, we examined such associations in the Vietnamese NSCLC cohort.

#### Gender

Gender status was available in 343 patients including 131 female and 212 male patients (1:1.6 ratio); the 7 patients with unknown sex were excluded from gender association analysis. We performed Chi-squared (χ²) test to investigate the association between patients’ gender and mutation prevalence. Consistent with previous studies, we found that *EGFR* mutations were more commonly detected in Vietnamese female patients than in male patients (48.1% versus 26.9%, p < 0.00001, Table 4). Conversely, *KRAS* mutation frequency was significantly higher in male than that in female patients (30.7% versus 9.2%, p < 0.0001, Table 4). Other driver genes including *NRAS*, *BRAF*, *ALK* and *ROS1* did not show any significant correlation

**Table 4:** Association between clinical factors and mutation frequencies of NSCLC driver genes.

Clinical characteristics		Total	*EGFR*					*KRAS*					*NRAS*				
			Not mutated		Mutated		p value	Not mutated		Mutated		p value	Not mutated		Mutated		p value
Sex	Female	131	68	51.9	63	48.1	**<0.0001**	119	90.8	12	9.2	**<0.00001**	131	100.0	0	0.0	NS
	Male	212	155	73.1	57	26.9		147	69.3	65	30.7		210	99.1	2	0.9	
	Unknown	7	3	42.9	4	57.1		5	71.4	2	28.6		7	100.0	0	0.0	
Age	<=61	174	112	64.4	62	35.6	NS	139	79.9	35	20.1	**<0.05**	172	98.9	2	1.1	NA
	>61	170	112	65.9	58	34.1		127	74.7	43	25.3		170	100.0	0	0.0	
	Unknown	6	2	33.3	4	66.7		5	83.3	1	16.7		6	100.0	0	0.0	
Smoking status	Yes	54	38	70.4	16	29.6	**<0.01**	38	70.4	16	29.6	**<0.0001**	54	100.0	0	0.0	NA
	No	131	63	48.1	68	51.9		122	93.1	9	6.9		131	100.0	0	0.0	
	Unknown	165	53	32.1	112	67.9		93	56.4	72	43.6		163	98.8	2	1.2	
Histology	ACC	241	155	64.3	86	35.7	**<0.05**	185	76.8	56	23.2	NS	240	99.6	1	0.4	NS
	SCC	25	21	84	4	16		19	76	6	24		24	96	1	4	
	Unknown	84	50	59.5	34	40.5		67	79.8	17	20.2		84	100.0	0	0.0	
**Clinical characteristic**		**Total**	***BRAF***					***ALK***					***ROS1***				
			**Not mutated**		**Mutated**		**p value**	**Not mutated**		**Mutated**		**p value**	**Not mutated**		**Mutated**		**p value**
Sex	Female	131	128	97.7	3	2.3	NS	120	91.6	11	8.4	NS	124	94.7	7	5.3	NS
	Male	212	207	97.6	5	2.4		201	94.8	11	5.2		208	98.1	4	1.9	
	Unknown	7	7	100.0	0	0.0		6	85.7	1	14.3		7	100.0	0	0.0	
Age	<=61	174	171	98.3	3	1.7	NS	157	90.2	17	9.8	**<0.05**	165	94.8	9	5.2	**<0.05**
	>61	170	165	97.1	5	2.9		164	96.5	6	3.5		168	98.8	2	1.2	
	Unknown	6	6	100.0	0	0.0		6	100.0	0	0.0		6	100.0	0	0.0	
Smoking status	Yes	54	54	100.0	0	0.0	**NA**	53	98.1	1	1.9	NS	54	100.0	0	0.0	NA
	No	131	127	96.9	4	3.1		122	93.1	9	6.9		131	100.0	0	0.0	
	Unknown	165	159	96.4	6	3.6		144	87.3	21	12.7		154	93.3	11	6.7	
Histology	ACC	241	235	97.5	6	2.5	NS	223	92.5	18	7.5	NA	236	97.9	5	2.1	NS
	SCC	25	24	96	1	4		25	100	0	0		24	96	1	4	
	Unknown	84	83	98.8	1	1.2		79	94.0	5	6.0		79	94.0	5	6.0	

N: total case number

n: number of particular group

%: percentage of particular cases in total number cases

Chi squared (χ²) test (sample size >5) or Fisher’s exact test (sample size< = 5) was performed to estimate p value.

AC: adenocarcinoma; SCC: squamous carcinoma; UN: unknown;

#### Age

Patient age was available in 344 patients, ranging from to 24 to 89 years old. When the median age (61 years) was used as a cutoff value, *KRAS* mutations were more frequently detected in elderly patients aged over 61 years than younger individuals (25.3% versus 20.1%, p < 0.05, Table 4). In contrast, the younger group showed higher prevalence of *ALK* (9.8% versus 3.5%, p < 0.05) and *ROS1* (5.2% versus 1.2%, p < 0.05) mutations (Table 4). There was no significant correlation between patient age and mutation prevalence of other driver genes including *EGFR*, *NRAS* and *BRAF* (Table 4).

#### Smoking status

Of the 185 cases with smoking status, we could detect a statistically significant correlation between *EGFR* and *KRAS* mutation prevalence and smoking status (Table 4). Our data indicated that non-smokers showed significantly higher frequency of *EGFR* mutation (51.9% versus 29.6%, p < 0.01) but lower frequency of *KRAS* mutation (6.9% versus 29.6%, p < 0.001) than smokers.

#### Histology

Adenocarcinoma (AC) were diagnosed in 241 patients, accounting for the most common histological type (81%), while squamous cell carcinoma (SCC) were identified in 25 cases (8.4%). We detected a significant association between histology type and *EGFR* mutation with higher prevalence in AC group than SCC group (35.7% versus 16%, p < 0.05) (Table 4).

## Discussion

NSCLC is the most common type of lung cancer with high rates of acquired somatic mutations^2^. Comprehensive profiling of clinically relevant mutations is of great importance in clinical practice for designing optimal targeted therapy as well as understanding drug resistance mechanisms^30,31^. Given the diversity of mutation constitution in different populations, the primary objective of our study is to examine the mutation profiles of major druggable driver genes in Vietnamese NSCLC patients. To this end, we performed targeted capture sequencing on 350 tumour tissue samples from Vietnamese patients with NSCLC and analyzed their genomic alterations in the six most common driver genes recommended by the ASCO and National Comprehensive Cancer Network for mutation testing in NSCLC patients^17^.

Our data showed that 66.3% of patients in the Vietnamese cohort harbour at least one alteration in the six tested driver genes. Our findings were consistent with our previous study using the same panel of genes and reporting a similar mutation rate of 63.6% in a smaller cohort of 59 Vietnamese NSCLC patients^32^. The mutation profiles of Vietnamese NSCLC patients also exhibited certain common features of NSCLC patients previously reported^33,34^. Firstly, mutations in *EGFR* and *KRAS* are the most common, accounted for more than 50% of total cases, while mutations in *ALK*, *BRAF*, *NRAS* and *ROS1* were rarer. This trend was also reported in a Chinese cohort by Zhuang *et al*.^33^ or in Caucasian populations by Campbell *et al*.^34^. Secondly, the mutation sites within the driver genes identified in this cohort were similar to those reported in other populations, including these most common mutation sites: *EGFR* exon 19 deletion (del19) and exon 21 (L858R)^35,36^, *KRAS* exon 2 (G12C)^37^, *BRAF* V600E^38^ and *ALK-EML4* fusion^39^. The frequencies of *ALK* (5.4%) and *ROS1* (2.9%) mutations determined in our study (Fig. 1) are comparable to previously published studies reporting the frequencies of 5.05 and 1%-2% for *ALK* and *ROS1*, respectively^40,41^. *EGFR* del19, *EGFR* L858R, *BRAF* V600E, *ALK*-*EML4* and *ROS1* fusion mutations in combined (139 cases) accounted for 39.7% of cases in the Vietnamese cohort. They were known as activating mutations and clinically proven to be sensitive to treatments with available TKI drugs^5,6,11,39^. Thus, our findings suggested that approximately 40% of Vietnamese patients would carry such mutations and therefore would benefit from available targeted drugs. Among 6 patients currently known to be on TKI therapy (Table 1), 5 cases carrying activating *EGFR* mutations (*EGFR* del19, *EGFR* L858R) and one case positive for both *EGFR* L858R and *ALK-EML4* fusion.

In contrast, some patients with activating *EGFR* mutations were found to develop an acquired resistance mutation, T790M^42,43^. Consistent with previous studies, we found that 7 out of 8 T790M cases were also positive for either L858R or del19 mutations and we suspected that these patients were under treatment with TKI drugs although we could not obtain treatment data for these cases. In addition to T790M, ins20 mutations were also known as resistant mutations^44,45^ and were detected in 8 cases in our cohort and most of them (6/8 cases) did not co-exist with any activating *EGFR* mutations L858R and del19, suggesting that ins20 mutations are likely primary inactivating mutations rather than acquired resistant mutations. Although a significant proportion of Vietnamese NSCLC patients were identified to carry *KRAS* mutations, drugs directly targeting *KRAS* mutated NSCLC are still under clinical evaluation^46^.

Although concomitant driver gene mutations in *EGFR*, *KRAS*, *BRAF* and *ALK* were initially reported to be mutually exclusive events in NSCLC patients^20,47^, we detected 14 cases (4%) harbouring concurrent alterations among the tested driver genes. These mutations might either coexist in the same tumour cell or belong to different tumour cell lines. The proportion of cases with co-mutations varied among studies. A recent study by Zhuang *et al*.^33^ involving a cohort of 3774 Chinese NSCLC patients reported a lower co-mutation rate of 1.67% in 5 tested driver genes (*EGFR*, *KRAS*, *ALK*, *ROS1* and *BRAF*) while 5% of patients in another cohort of 1,000 NSCLC patients at The NCI’s Lung Cancer Mutation Consortium were reported to harbour concomitant driver gene mutations^48^. However, these studies together with our study consistently reported the *EGRF*/*KRAS* (6/14 cases in our study) as the most common co-mutation event in NSCLC patients^33,48^. The identification of patients with such co-mutations is of clinical importance since these concurrent mutations represent a distinct subset of patients and may have significant impact on treatment outcomes. In this regard, previous studies showed that patients carrying *EGFR*/*ALK* co-mutations varied in their sensitivity to and that the choice between these two classes of TKI drugs as first-line treatment for these patients is still being debated^49^. Hence, further studies are required to investigate clinical activity and drug sensitivity of different co-mutation subsets in order to develop suitable treatment approaches.

To identify Vietnamese-specific mutation profiles in NSCLC patients, we selected patients with comparable histology (AC) and tumour stage (stage III-IV) from the East Asian cohort (China)^23^ and the MSK-IMPACT cohort mainly consisting of Caucasia patients^21,22^. The prevalence of *EGFR* mutations among Vietnamese NSCLC patients was markedly lower than East Asia cohort (37.7% versus 73.4%, p < 0.00001, Table 3) but significantly higher than MSK-IMPACT cohorts (37.7% versus 29.1%, p < 0.05, Table 3), confirming previous reports that *EGFR* mutations are more prevalent in Asian patients than in Caucasian patients^50^. Of note, the percentage of Vietnamese patients with *KRAS* mutation including those with concurrent mutations was comparable to the MSK-IMPACT cohort but significantly higher than the published data in East Asian (21.4% versus 9.1%, p < 0.0001). Hence, our results demonstrated that NSCLC patients from Vietnamese population exhibit a unique mutation constitution, suggesting that ethnic composition might contribute to the observed variation in mutation profiles. Furthermore, Nguyen *et al*.^32^ reported a remarkably higher frequency of *KRAS* mutations in Vietnamese patients living in Vietnam than in those living in the USA (24.4% versus 4.5%), suggesting that geographic and socioeconomic disparities might also contribute to the variation in mutation frequencies in different cohorts. Although the clinical significance and mechanisms driving these variations are unclear, the unique mutation profiles should be taken into consideration for prioritizing research programs aiming to develop new treatment strategies for Vietnamese NSCLC patients.

*KRAS* mutations were detected in 30% of NSCLC patients who were non-responsive to TKI treatment^51^. Hence, the high prevalence of *KRAS* mutations in the Vietnamese NSCLC cohort might have negative impacts on clinical outcomes. However, the use of *KRAS* mutation status as a negatively predictive marker of TKI therapy remains controversial due to inconsistent results obtained from subsequent meta-analysis studies^52–55^. Interestingly, *KRAS* mutations, particularly the most prevalent subtype G12C, when co-existing with PD-L1 expression in patients’ tumour were shown to have poor prognosis^56^, supporting the potential benefit of *KRAS* mutation testing in selecting patients for immunotherapy using check-point inhibitor.

We further investigated correlations between mutation prevalence and major patients’ clinical characteristics. Consistent with previous studies^25,50^, we observed that *EGFR* mutations were more prevalent in female patients, non-smoker and those with histological subtype of AC. Unlike *EGFR* mutations, *KRAS* mutations were more commonly detected in male patients and showed significant correlations with patients’ age, with higher prevalence in elder patients. It is possible that the high prevalence of *KRAS* mutations in Vietnamese male patients may be responsible for their higher mortality rate. Previous studies reported that *KRAS* mutations more frequently arise in smokers than in non-smokers^25,57^. Consistently, we observed such correlation in our study, indicating that the high frequency of *KRAS* mutation in our cohort could be attributable to high prevalence of smoking in Vietnam population. In addition, we found that *ALK* and *ROS1* rearrangement mutations were more common in younger patients as compared to elderly patients, which is consistent with previous studies^28,29^. Take together, our data revealed several significant correlations between driver gene mutation prevalence and patients’ clinical characteristics.

There are a few limitations in our study. Firstly, although the panel of driver genes used in this study was chosen based on ASCO guidelines^5^, we did not take into account mutations in other driver genes such as *PIK3CA*^58^, *AKT1*^59^ and *ERBB2*^60^, previously reported to co-exist with those detected mutations and possibly having significant clinical impacts. Secondly, when comparing the mutation profiles of the Vietnamese cohorts with MSK-IMPACT and East Asian cohort, we selected patients with AC in late stages (stage III-IV) to exclude the confounding effect of histological subtypes and tumour stage that are varied among three cohorts. However, there were still differences in patients’ age at diagnosis and gender ratio between the Vietnamese and the other two cohorts, which were identified be significant factors associated with *EGFR* and *KRAS* mutation prevalence, thus might have impact on the analysis of the mutation profiles. Future large-scale studies are required to assess whether these confounders contribute to the variations in mutation profile between the of Vietnamese population and other races.

In conclusions, our study revealed the mutation profiles of multiple driver genes in the largest cohort of NSCLC patients in Vietnam to date. Our data highlighted several subsets of Vietnamese NSCLC patients carrying specific mutations that would benefit from future studies to provide more suitable treatment options.

## Material and Methods

### Tumour tissues

We studied 350 formalin fixed, paraffin-embedded tumour specimens from NSCLC patients treated at Pham Ngoc Thach hospital, Cho Ray hospital, Ha Noi Oncology hospital and Vietnam National cancer hospital. The tumour-rich areas of the tissues that contain at least 20% of tumour cells identified by a hematoxylin and eosin staining were micro-dissected. Written informed consents were obtained from all patients. Clinical characteristics of all patients were summarised in Table S1. This study was approved by The Ethic Committee of University of Medicine and Pharmacy at Ho Chi Minh City, Vietnam (Ethic number: 027/DHYD-HD) and The Medical Genetics Institute. All methods were performed in accordance with the relevant guidelines and regulations.

### DNA isolation

DNA was extracted from FFPE samples using QIAamp DNA FFPE Tissue Kit (Qiagen, USA) following the manufacturer’s instructions and then quantified using the QuantiFlour dsDNA system (Promega, USA).

### Massively parallel sequencing

DNA fragmentation and library preparation were performed using the NEBNext Ultra II FS DNA library prep kit (New England Biolabs, USA) following the manufacturer’s instructions. DNA library concentrations were quantified with a QuantiFlour dsDNA system (Promega, USA). Equal amounts of libraries (150 ng per sample) were pooled together and hybridized with xGen Lockdown probes for six targeted genes *EGFR, KRAS, NRAS, BRAF, ALK and ROS1* (IDT DNA, USA). For *ALK* and *ROS1*, customized probes (Table S3) for intron regions were designed and mixed with probes for exon regions at equal concentration. Sequencing was run using NextSeq. 500/550 High output kits v2 (150 cycles) on Illumina NextSeq. 550 system (Illumina, USA) with minimum target coverage of 100×. In cases where the mean coverage in the targeted regions is lower than 100×, extra sequencing was performed to increase the mean coverage to the expected range. The mean coverage in the target regions for all samples is approximately 129×.

### Variant calling using Mutect2 and Factera

Each FFPE sample was barcoded with dual indexes in the P7 and P5 primer. The PE reads were generated by bcl2fastq package (Illumina) and aligned to human genome (hg38) using BWA package^61^. Duplicate reads were marked using MarkDuplicates from Picard tools (http://broadinstitute.github.io/picard/). Somatic variants were called using Mutect2 package^62^. A custom pipeline with call to BWA, Picard, and Samtools packages were built to perform the above-mentioned analysis steps^63^. For detection of *ALK* and *ROS1* rearrangement, fusion variant calling was analyzed using Factera v1.4.4 with default parameters^64^.

### ddPCR method

A four-step ddPCR procedure was performed using reagents and equipment from Bio-Rad (unless otherwise stated) following the manufacturer’s instruction^65^. Briefly, the PCR mix was first prepared by mixing 1 × ddPCR Supermix for Probes, primers and probes (IDTDNA) and DNA template (0.8 or 1.6 ng). Next, 20 µl of the PCR mix was transferred into the Droplet Generator DG8TM Cartridge followed by 70 μl of the Droplet Generation Oil before placing in a QX100TM Droplet Generator to generate droplets. Subsequently, the droplets were transferred to a 96-well plate before placing in a thermal cycler (C1000 Touch, Bio-Rad) for PCR amplification. The PCR thermal program was performed as follows: 95 °C for 10 min, then 40 successive cycles of amplification (94 °C for 30 sec; 55 °C for 60 sec) and 98 °C for 10 min. Lastly, the droplet reading was acquired by the QX 200 Droplet reader and analyzed using the QuantaSoft Software. Positive and negative droplets are assigned based on the fluorescence threshold that was set as previously described by Deprez *et al*.^66^.

To detect T790M and L858R mutations in exon 20 and 21 of the *EGFR* gene, one reaction of ddPCR was used with two sets of primers and probes as follows: T790M primer F-GCCTGCTGGGCATCTG; T790M primer R-TCTTTGTGTTCCCGGACATAGTC; T790M mutation probe FAM- ATGAGCTGC**A**TGATGAG-ZEN/3’IBFQ; L858R primer F-GCAGCATGTCAAGATCACAGATT; L858R primer R-CCTCCTTCTGCATGGTATTCTTTCT; L858R mutation probe HEX-AGTTTGGCC**C**GCCCAA- ZEN/3’IBFQ. For detection of 15 deletion sites in exon 19 (del19) of the *EGFR* gene, a commercially available ddPCR reaction (Bio-rad) was used (ddPCR™ EGFR Exon 19 Deletions Screening Kit #12002392).

### Statistical analysis

Pearson’s chi-squared (χ²) test (sample size >5) or Fisher’s exact test (sample size< = 5) was performed on the web page ‘Social Science Statistics’ (http://www.socscistatistics.com) to assess the association between two categorical variables (Tables 3 and 4). Bonferroni correction was applied when multiple comparisons were performed (Table 3).

## Supplementary information

Supplementary information.
