# Supplementary material for: Actionable Mutation Profiles of Non-Small Cell Lung Cancer patients from Vietnamese population
Source: Sci Rep. 2020 Feb 17;10:2707. doi: 10.1038/s41598-020-59744-3 (PMC7026432; doi:10.1038/s41598-020-59744-3)
Supplement: Supplementary file 1 — Supplementary information. [file 41598_2020_59744_MOESM1_ESM.pdf]

Actionable Mutation Profiles of Non-Small Cell Lung Cancer patients from Vietnamese population

Anh-Thu Huynh Dang, Vu-Uyen Tran, Thanh-Truong Tran , Hong-Anh Thi Pham, Dinh-Thong Le, Lam Nguyen, Ngoc-Vu Nguyen, Thai-Hoa Thi Nguyen, Chu Van Nguyen, Ha Thu Le, Mai-Lan Thi Nguyen, Vu Thuong Le, Phuc Huu Nguyen, Binh Thanh Vo, Hong-Thuy Thi Dao, Luan Thanh Nguyen, Thien-Chi Van Nguyen, Quynh-Tram Nguyen Bui, Long Hung Nguyen, Nguyen Huu Nguyen, Quynh-Tho Thi Nguyen, Truong Xuan Le, Thanh-Thuy Thi Do, Kiet Truong Dinh, Han Ngoc Do, Minh-Duy Phan, Hoai-Nghia Nguyen, Le Son Tran, Hoa Giang

Table S1 Patients's clinical characteristics NA: unknown cases; AC: adenocarcinoma; SCC: squamous cell carcinoma; LL: left lung; RL: right lung; BC: bronchus; L: lung;

Case No.	Patient ID	Sex	Age	Smoking status	Histology	Tumor Location	Stages	Treatment details
1	F0008	M	49	No	AC	LL	IV	No
2	F0010	F	64	No	AC	RL	NA	NA
3	F0014	M	71	NA	SCC	BC	IIIA	No
4	F0017	F	53	No	AC	BC	II	No
5	F0019	F	64	No	AC	LL	IIIA	No
6	F0020	M	71	NA	AC	RL	IV	No
7	F0022	M	72	NA	AC	RL	IV	No
8	F0025	M	47	No	AC	RL	IV	No
9	F0028	M	59	Yes	AC	LL	IV	NA
10	F0033	M	83	NA	NA	NA	NA	NA
11	F0034	M	69	NA	AC	LL	NA	No
12	F0036	F	80	Yes	AC	RL	IV	NA
13	F0038	F	57	No	AC	NA	IV	No
14	F0039	M	57	NA	NA	L	IV	surgical resection
15	F0041	F	71	No	AC	L	IV	NA
16	F0043	M	51	NA	AC	BC	IV	No
17	F0045	M	55	NA	AC	RL	IV	No
18	F0046	M	50	NA	Others	LL	IV	No
19	F0047	NA	70	Yes	AC	L	III	NA
20	F0048	M	60	NA	AC	RL	III	NA
21	F0049	M	52	NA	AC	RL	IVA	No
22	F0050	M	64	Yes	SCC	RL	IV	NA
23	F0051	M	76	NA	AC	L	IV	NA
24	F0052	M	70	NA	AC	L	IV	No
25	F0054	M	64	NA	AC	LL	IV	surgical resection and chemotherapy
26	F0055	M	69	NA	SCC	L	IV	No
27	F0057	F	NA	No	NA	L	III	No
28	F0058	M	79	NA	AC	RL	II	No
29	F0059	M	56	Yes	NA	L	IV	Chemotherapy
30	F0060	M	70	NA	AC	BC	IV	NA
31	F0061	M	65	NA	AC	RL	IV	No
32	F0062	M	48	NA	SCC	BC	IV	No
33	F0063	M	60	Yes	AC	L	IV	No
34	F0064	M	63	Yes	AC	RL	IV	No
35	F0065	M	50	NA	AC	Nodule	IV	No
36	F0070	M	73	NA	AC	RL	IV	No

37	F0071	M	71	Yes	AC	LL	IV	No
38	F0072	F	44	No	AC	RL	T2	surgical resection
39	F0073	M	67	No	AC	LL	IV	No
40	F0079	M	56	NA	AC	L	IV	No
41	F0081	F	62	No	AC	BC	IV	No
42	F0082	M	69	No	AC	LL	IIIB	NA
43	F0083	F	59	No	AC	BC	IV	NA
44	F0084	M	58	Yes	AC	Nodule	NA	NA
45	F0085	M	31	Yes	AC	L	IV	No
46	F0086	M	53	NA	AC	BC	IV	NA
47	F0088	M	76	NA	AC	LL	I	No
48	F0089	F	66	No	AC	RL	IV	No
49	F0090	F	68	No	AC	BC	IV	NA
50	F0091	M	51	NA	AC	L	III	No
51	F0093	M	64	NA	AC	LL	IV	No
52	F0094	M	56	NA	AC	BC	IV	No
53	F0096	F	47	No	AC	Liver	IV	No
54	F0098	M	66	Yes	AC	nodule	I	No
55	F0100	M	65	Yes	NA	NA	IV	No
56	F0101	F	55	No	AC	L	IV	No
57	F0102	F	43	No	AC	LL	IV	NA
58	F0103	M	64	NA	AC	BC	III	NA
59	F0104	M	48	NA	AC	L	IV	NA
60	F0105	M	70	NA	AC	NA	IV	NA
61	F0106	M	67	NA	AC	BC	IV	Chemotherapy and radiation therapy
62	F0107	F	59	No	AC	L	IV	No
63	F0108	M	61	NA	AC	BC	IV	No
64	F0110	M	68	NA	NA	L	IV	No
65	F0111	F	57	No	NA	L	IV	No
66	F0112	M	63	Yes	AC	BC	IV	No
67	F0114	M	70	NA	NA	L	IV	NA
68	F0115	M	66	Yes	AC	RL	IV	No
69	F0116	M	36	NA	AC	L	IV	No
70	F0117	M	45	Yes	AC	LL	IIIB	No
71	F0120	M	52	Yes	AC	RL	IV	No
72	F0121	F	65	Yes	NA	L	IV	Chemotherapy
73	F0122	M	82	NA	NA	L	IV	NA
74	F0123	M	42	Yes	NA	RL	IV	No
75	F0124	F	69	No	NA	NA	IV	No
76	F0125	M	66	Yes	AC	Nodule	IV	NA
77	F0126	NA	NA	NA	NA	L	NA	NA
78	F0127	M	66	Yes	AC	L	IV	No
79	F0129	F	78	No	AC	RL	IV	NA
80	F0131	M	54	Yes	AC	BC	IV	NA

81	F0133	F	32	No	NEC	L	IV	No
82	F0135	M	27	NA	NA	RL	IIb	No
83	F0137	F	59	No	AC	BC	IV	No
84	F0140	M	59	NA	AC	L	IV	No
85	F0142	F	66	No	AC	L	IV	No
86	F0143	M	71	Yes	AC	L	IV	No
87	F0144	F	57	No	AC	LL	IV	No
88	F0145	NA	NA	NA	NA	L	NA	NA
89	F0146	NA	NA	NA	NA	L	NA	NA
90	F0148	M	49	NA	NA	L	IV	No
91	F0151	F	71	No	AC	L	IV	No
92	F0152	NA	NA	NA	NA	L	NA	NA
93	F0153	NA	NA	NA	NA	L	NA	NA
94	F0154	F	69	No	NA	L	IV	No
95	F0155	F	56	No	NA	L	IV	No
96	F0173	M	69	NA	AC	L	IV	No
97	F0174	M	78	NA	AC	LL	IV	NA
98	F0176	M	67	Yes	NA	L	IV	No
99	F0179	M	72	NA	NA	L	IV	No
100	F0180	F	62	No	AC	RL	IV	No
101	F0181	F	76	No	AC	RL	IV	No
102	F0182	M	66	Yes	NA	L	IV	No
103	F0184	F	74	No	AC	Liver	IV	Chemotherapy
104	F0185	M	60	NA	SCC	RL	IIIB	No
105	F0186	M	65	NA	NA	L	IV	NA
106	F0187	F	60	No	AC	LL	IV	No
107	F0188	M	69	NA	AC	RL	IV	No
108	F0190	F	57	No	SCC	LL	IV	No
109	F0191	F	35	No	AC	BC	IV	No
110	F0193	M	68	NA	NA	L	IV	No
111	F0195	M	62	NA	AC	LL	IV	No
112	F0196	M	67	NA	AC	BC	IV	No
113	F0197	M	61	NA	AC	BC	IIIB	No
114	F0198	M	59	NA	AC	L	IV	No
115	F0199	M	81	NA	AC	L	IV	No
116	F0200	M	61	Yes	AC	BC	IV	No
117	F0201	M	68	Yes	AC	L	IIIB	No
118	F0202	M	64	NA	SCC	RL	IV	No
119	F0203	M	59	NA	AC	LL	IV	No
120	F0206	M	79	NA	AC	L	IV	No
121	F0207	F	53	No	AC	BC	IV	No
122	F0208	F	58	No	AC	BC	IV	No
123	F0209	M	71	NA	AC	LL	IV	No
124	F0211	M	54	NA	SCC	RL	NA	Yes
125	F0212	M	74	NA	AC	BC	IV	Yes
126	F0214	M	62	NA	AC	BC	IV	No

127	F0215	F	55	No	AC	L	IV	TKI (13 months)
128	F0216	M	64	NA	AC	LL	IV	No
129	F0217	M	59	NA	AC	nodule	IV	No
130	F0218	F	60	No	AC	L	IIIB	No
131	F0219	M	56	NA	NA	L	NA	TKI (ongoing)
132	F0221	F	55	No	AC	Pleural	IV	No
133	F0222	F	71	No	AC	BC	IV	No
134	F0224	F	50	No	NA	RL	IV	No
135	F0225	M	61	Yes	NA	LL	IV	No
136	F0226	M	57	NA	AC	BC	IV	No
137	F0227	F	49	No	AC	L	IV	No
138	F0228	F	45	No	NA	BC	IV	No
139	F0229	F	68	No	AC	L	NA	No
140	F0231	M	72	NA	AC	RL	IV	No
141	F0232	M	71	NA	AC	BC	IV	No
142	F0233	M	56	Yes	AC	LL	IV	No
143	F0234	M	43	NA	NA	L	NA	No
144	F0235	F	69	No	SCC	LL	IV	No
145	F0236	M	53	NA	AC	L	IV	No
146	F0237	F	42	Yes	AC	BC	IV	No
147	F0238	F	79	No	NA	L	IV	No
148	F0239	F	64	Yes	AC	RL	IV	No
149	F0240	M	73	Yes	NA	L	II	No
150	F0241	M	58	NA	AC	LL	IV	No
151	F0242	M	43	NA	AC	RL	IV	No
152	F0243	M	42	NA	AC	BC	NA	No
153	F0244	M	49	NA	NA	RL	NA	No
154	F0245	M	59	NA	AC	LL	IV	No
155	F0246	M	70	NA	NA	RL	IIIB	No
156	F0247	M	56	NA	NA	L	NA	No
157	F0249	M	67	NA	AC	LL	IV	No
158	F0250	M	65	NA	AC	BC	III	No
159	F0251	M	49	NA	AC	BC	IV	No
160	F0252	M	62	NA	NEC	nodule	IV	No
161	F0254	M	61	NA	AC	BC	IV	No
162	F0256	M	67	NA	AC	BC	IV	No
163	F0258	F	59	No	NA	BC	IIIB	No
164	F0259	M	75	NA	NA	LL	NA	No
165	F0260	M	67	NA	SCC	BC	II	NA
166	F0261	M	70	Yes	NA	L	IV	No
167	F0262	M	60	Yes	AC	L	IV	No
168	F0263	M	89	NA	SCC	L	IV	No
169	F0264	M	60	NA	AC	L	IV	No
170	F0265	M	45	NA	NA	L	IV	No
171	F0267	M	62	NA	AC	L	IIIb	No
172	F0268	M	57	NA	AC	L	IV	No

173	F0269	M	52	NA	AC	BC	NA	No
174	F0270	F	59	No	AC	LL	IV	TKI
175	F0272	F	67	No	AC	RL	IV	No
176	F0273	M	59	NA	NA	RL	NA	No
177	F0274	M	58	NA	NA	BC	NA	No
178	F0275	M	65	NA	AC	BC	NA	No
179	F0276	M	73	Yes	AC	RL	IV	No
180	F0277	M	71	NA	SCC	BC	NA	No
181	F0278	M	59	NA	NA	BC	NA	No
182	F0279	F	63	No	AC	L	IV	No
183	F0280	F	46	No	AC	nodule	IV	surgical resection
184	F0281	F	50	No	SCC	RL	IV	No
185	F0282	M	65	NA	NA	LL	NA	No
186	F0283	F	49	No	AC	L	IV	No
187	F0284	M	49	NA	AC	L	IV	No
188	F0285	M	64	NA	AC	LL	IV	No
189	F0287	F	57	No	AC	RL	IV	No
190	F0288	M	62	NA	AC	BC	NA	No
191	F0289	M	65	NA	SCC	LL	NA	No
192	F0290	F	46	No	NA	NA	IV	No
193	F0292	M	65	Yes	NA	NA	III	No
194	F0293	F	62	No	AC	BC	IV	No
195	F0294	M	61	NA	AC	LL	IV	No
196	F0295	F	68	No	AC	LL	IV	TKI (ongoing)
197	F0297	M	65	NA	SCC	LL	NA	No
198	F0298	F	57	No	NA	RL	IV	No
199	F0300	F	60	No	AC	LL	IV	No
200	F0301	M	59	Yes	AC	RL	IV	No
201	F0302	F	59	No	NA	RL	IV	No
202	F0304	M	42	NA	AC	LL	IV	No
203	F0305	F	67	No	AC	BC	IV	No
204	F0306	M	71	No	AC	BC	III	No
205	F0307	M	66	NA	NA	RL	IV	No
206	F0308	F	72	No	AC	L	III	No
207	F0309	M	60	Yes	AC	L	IV	Yes
208	F0310	F	74	No	AC	RL	IV	No
209	F0311	F	46	No	AC	RL	IV	No
210	F0312	M	55	NA	NA	L	IV	No
211	F0313	F	63	No	AC	L	IV	No
212	F0314	F	57	No	AC	RL	IV	No
213	F0315	M	59	NA	AC	L	IV	No
214	F0316	M	61	Yes	AC	Brain	IV	No
215	F0317	M	61	NA	NA	L	IV	No
216	F0319	F	34	No	AC	BC	IV	NA
217	F0320	F	57	No	SCC	RL	IV	No
218	F0321	M	53	NA	AC	RL	IV	Yes

219	F0322	M	48	NA	AC	L	IV	No
220	F0323	F	53	No	AC	L	IV	No
221	F0324	F	68	No	AC	LL	IV	No
222	F0326	M	67	Yes	AC	RL	IIIB	No
223	F0327	M	75	Yes	AC	L	III	No
224	F0328	F	34	No	AC	BC	IV	No
225	F0329	M	53	No	AC	RL	IIIB	No
226	F0330	M	79	NA	NA	LL	NA	No
227	F0331	F	65	No	NA	BC	IV	No
228	F0333	M	54	NA	NA	BC	NA	No
229	F0334	M	64	NA	AC	BC	NA	No
230	F0335	M	60	No	SCC	L	II	No
231	F0336	F	62	No	AC	RL	IVA	No
232	F0337	M	82	Yes	AC	BC	IVA	No
233	F0338	M	51	Yes	AC	BC	IIIC	No
234	F0339	F	64	No	NA	L	IV	No
235	F0340	M	42	NA	AC	BC	IV	No
236	F0341	F	71	No	AC	LL	IV	No
237	F0343	F	47	Yes	NA	Pleural	IV	No
238	F0344	M	71	NA	AC	L	IV	No
239	F0346	F	49	No	AC	RL	IV	No
240	F0349	M	77	NA	AC	LL	II	No
241	F0350	M	63	No	AC	RL	IV	No
242	F0352	F	42	No	NA	L	IV	No
243	F0353	F	39	No	AC	BC	IV	No
244	F0354	M	60	NA	NA	L	NA	No
245	F0355	M	60	NA	SCC	LL	IV	Chemotherapy
246	F0356	F	55	No	NA	BC	IV	No
247	F0357	M	59	NA	AC	nodule	IV	No
248	F0359	F	54	No	AC	BC	II	No
249	F0360	M	50	NA	AC	BC	IIIb	No
250	F0361	F	66	No	AC	BC	IV	No
251	F0362	M	60	NA	NA	BC	NA	No
252	F0363	F	44	No	AC	L	IV	No
253	F0365	M	56	NA	NA	L	NA	No
254	F0366	F	60	No	AC	LL	IV	No
255	F0367	M	66	NA	NA	L	NA	No
256	F0368	M	60	NA	NA	L	NA	No
257	F0369	F	59	No	SCC	L	IV	No
258	F0370	F	54	No	AC	L	IV	No
259	F0371	M	61	NA	NA	L	NA	No
260	F0372	F	67	No	AC	RL	IIIB	No
261	F0373	M	68	NA	AC	L	IV	No
262	F0374	F	61	No	NA	BC	NA	No
263	F0375	M	47	Yes	NA	BC	IV	No
264	F0376	M	53	NA	Others	LL	IV	No

265	F0377	F	77	No	NA	L	NA	No
266	F0378	M	78	NA	NA	L	NA	No
267	F0379	F	70	No	AC	LL	IV	No
268	F0380	M	66	NA	SCC	LL	IV	No
269	F0381	F	70	No	AC	RL	IV	No
270	F0382	M	66	Yes	AC	LL	IV	No
271	F0383	M	69	NA	AC	L	IV	No
272	F0384	M	86	NA	AC	L	IV	No
273	F0385	M	62	NA	AC	BC	NA	No
274	F0386	M	67	NA	AC	BC	NA	No
275	F0388	F	32	No	AC	RL	IV	No
276	F0389	M	58	Yes	AC	RL	IV	No
277	F0390	F	46	No	AC	NA	IV	No
278	F0391	NA	56	NA	AC	L	IV	No
279	F0392	F	57	No	AC	L	IV	No
280	F0393	F	66	No	NA	L	NA	No
281	F0394	M	53	NA	AC	RL	IV	No
282	F0395	F	49	No	AC	L	IIB	No
283	F0396	M	58	NA	AC	B	IV	No
284	F0397	F	75	No	AC	LL	IV	No
285	F0398	M	66	NA	AC	L	IV	No
286	F0400	F	60	No	AC	RL	IV	No
287	F0401	F	64	No	AC	L	IIIB	No
288	F0402	F	47	No	AC	RL	IV	Chemotherapy
289	F0403	F	69	No	AC	LL	IV	No
290	F0404	M	61	NA	AC	RL	IV	No
291	F0405	M	56	Yes	AC	L	IV	No
292	F0406	M	72	NA	Others	RL	IV	No
293	F0407	F	69	No	AC	LL	IV	No
294	F0411	F	36	No	AC	RL	IV	No
295	F0412	F	45	No	AC	NA	IV	No
296	F0413	M	70	No	AC	NA	IV	No
297	F0414	M	65	NA	AC	NA	IVa	No
298	F0415	M	43	No	AC	NA	IV	Chemotherapy
299	F0416	F	65	No	AC	RL	IV	Chemotherapy
300	F0417	M	53	NA	AC	LL	IV	No
301	F0418	M	60	Yes	AC	NA	IV	No
302	F0419	M	62	Yes	AC	NA	III	No
303	F0421	M	61	Yes	AC	NA	IV	No
304	F0422	F	35	No	SCC	LL	IV	No
305	F0423	M	69	NA	AC	LL	IIIb	TKI (Iressa, 6 months)
306	F0424	F	66	No	SCC	RL	IIIB	No
307	F0426	M	71	NA	AC	NA	NA	No
308	F0427	M	72	NA	NA	NA	NA	No
309	F0428	F	64	No	AC	NA	IV	No

310	F0429	F	56	NA	AC	NA	IV	No
311	F0431	F	81	No	AC	LL	IV	No
312	F0433	F	67	No	AC	LL	IV	No
313	F0434	F	62	NA	AC	BC	IV	No
314	F0435	M	42	NA	AC	NA	IV	NA
315	F0436	M	54	NA	AC	L	IVB	No
316	F0437	F	57	No	AC	Brain	IV	surgical resection
317	F0438	M	69	NA	AC	BC	IV	No
318	F0439	M	63	NA	AC	L	IV	No
319	F0440	F	60	NA	NA	NA	IV	No
320	F0441	F	46	NA	AC	L	IV	No
321	F0442	F	58	No	NA	L	IV	No
322	F0443	F	70	NA	AC	RL	IV	Yes
323	F0445	M	48	NA	AC	BC	IV	No
324	F0446	F	52	No	NA	NA	NA	No
325	F0447	F	58	No	AC	NA	IV	TKI (tarceva)
326	F0448	M	62	NA	AC	NA	IV	No
327	F0450	F	65	No	AC	RL	III	No
328	F0451	F	62	No	AC	RL	IV	No
329	F0452	F	41	No	AC	LL	IV	No
330	F0453	F	72	No	AC	RL	IV	No
331	F0455	M	63	Yes	AC	NA	III	Chemotherapy
332	F0456	M	60	NA	SCC	NA	IV	NA
333	F0457	M	75	NA	SCC	NA	IV	No
334	F0459	M	55	NA	AC	RL	III	NA
335	F0460	F	79	No	NA	NA	IV	No
336	F0461	F	65	No	AC	NA	IV	No
337	F0462	F	44	No	NA	NA	IV	No
338	F0463	M	54	NA	AC	L	IV	NA
339	F0464	M	65	NA	NA	NA	NA	NA
340	F0465	M	80	Yes	AC	NA	IV	No
341	F0466	F	45	No	AC	LL	IV	Chemotherapy
342	F0467	F	62	No	AC	L	IV	No
343	F0468	M	52	NA	AC	NA	NA	No
344	F0469	F	65	No	AC	NA	IV	No
345	F0470	M	60	NA	SCC	RL	IV	No
346	F0471	M	62	Yes	AC	LL	IV	No
347	F0472	M	73	NA	NA	NA	NA	NA
348	F0473	F	57	No	NA	NA	IV	No
349	F0475	M	82	Yes	AC	NA	IIIB	No
350	F0476	M	62	NA	NA	NA	NA	No

Table S2 *EGFR* mutation results determined by massively parallel sequencing and ddPCR

(-): mutations not detected

Case No.	Sample ID	Massively Parallel Sequencing		ddPCR	
		Mutation	VAF (%)	Mutation	VAF (%)
1	LBL015	*EGFR* L858R	65	*EGFR* L858R	57.0
2	LBL017	*EGFR* L858R	90	*EGFR* L858R	82.3
3	L10055	*EGFR* L858R	55	*EGFR* L858R	28.0
4	L10019	*EGFR* del19	11	*EGFR* del19	12.7
5	L10021	(-)		*EGFR* del19	0.5
6	L10036	*EGFR* del19	44	*EGFR* del19	24.8
7	L10072	*EGFR* del19	50	*EGFR* del19	58.2
8	L10076	*EGFR* del19	34	*EGFR* del19	40.9
9	LBL021	(-)		*EGFR* del19	3.9
10	LBL033	*EGFR* del19	20	*EGFR* del19	24.6
11	L10022	*EGFR* L858R<br>*EGFR* T790M	37<br>43	*EGFR* L858R<br>*EGFR* T790M	22.3<br>28.2
12	LBL026	*EGFR* del19	23	*EGFR* del19	27.6
13	LBL030	*EGFR* del19	10	*EGFR* del19	6.8
14	LBL001	(-)		(-)	
15	LBL002	(-)		(-)	
16	LBL003	(-)		(-)	
17	LBL004	(-)		(-)	
18	LBL005	(-)		(-)	
19	LBL006	(-)		(-)	
20	LBL007	(-)		(-)	
21	LBL008	(-)		(-)	
22	LBL009	(-)		(-)	
23	LBL012	(-)		(-)	
24	LBL013	(-)		(-)	
25	LBL014	(-)		(-)	
26	LBL016	(-)		(-)	
27	LBL020	(-)		(-)	
28	LBL022	(-)		(-)	
29	LBL023	(-)		(-)	
30	LBL024	(-)		(-)	
31	LBL025	(-)		(-)	
32	LBL027	(-)		(-)	
33	LBL028	(-)		(-)	
34	LBL029	(-)		(-)	
35	LBL031	(-)		(-)	
36	LBL034	(-)		(-)	
37	LBL036	(-)		(-)	
38	LBL037	(-)		(-)	
39	LBL040	(-)		(-)	

40	LBL041	(-)		(-)	

Table S3 Probe sequences for detecting *ALK* and *ROS1* fusions

Number	ID	Sequence
1	ALK19_1	GGTGAGTGCACAGAGCCCCAGGGACTCCCAAGGGGGCAGGAAGGCAGGACTGAATAGTGTCTCAGGCTGTGCCACAGGTGCCAAGGTGTCACTTCGTTATGCTAGTCCCTGGAATTGGGT
2	ALK19_2	GGGGTGGTGATTAGGGCAGCCCAGGCCAAGCCAAAACGGAAGCTCCCAACCTTCCCCCCACCAGAGCAGCTGCAGTTCCCTGAGGAGCCCCTGATTCTGCACCTCAGCCCCGTGTGTATC
3	ALK19_3	CTCCTGGCTGATCAGGGGGTGGGGAGCTCCTTCAGTGTCCATCACGATGGTGAAAGCTCGCCCCCACCCCTAGACGTCACTTCTAGCTCCCACATGCTTCCACCGGCGCAGCTCCTGTTT
4	ALK19_4	GGCTCCCACCCTATGTAATGCACTAGCCCACTCTTCCCCAAACCAGCCCTCCACCACCCTCCAGGCAGAGAGATAGGAAAATCGGTTTCTGAGTATATTTCTGTTCAGCCTGTGAGCCAA
5	ALK19_5	GGTGAGCTGACCTGCAGGTCACAGAGAACTCAGTGTGGTCCCAACCAGCTCTTACTGCTGGCAGAGACATGCCCAGGACAGATGGGCAGAGGCTTGAAAAGGGCAGAGGGAAAGGCTCTT
6	ALK19_6	GAGAGCCCTCGCAGGCCAGGCCCCTGCAGGCAAAGGGATCTGCCGGTAGAAGGGAGATGGCAGCACACACTGTGTCCCCATATGGTGCCATCCCTCAAAGGGACAGGATAATAGGAGCTA
7	ALK19_7	ACACTTGTTGCATGGTTACTACGTGCTCGGCAATTTACACATTTCAATTCATTCGATCCTCAGGTAACCCTAATCTGATCACGGTCGGTCCATTGCATAGAGGAGGGAACTGAGCACATA
8	ALK19_8	GCGGGTGACTCATTTGCCCTGGCCCATGTGTTGGGGGGCTGGGCTTTACACACAGAATCTACCCACTGAATCACAATTTTGTTCTGGCTTCCATGGAGTTTGCCTTCCAGAACATCCTCA
9	ALK19_9	CATGTAGGAGTGATAATGGTCACTCACATTGGTAGAGCTCTTTAGGATTTTTCAAAACCATTTTATGTTGGTGAATTCATTTCATTGACAACCCTAGAGGGTGGGGAGTGGCAGTGGTTA
10	ALK19_10	GGGAAACAGGGCAGGAGTTACCATCCCTGCCTACAGAGAGGGAAACTGCAGTCCAAAGAGGTCCTGTGACCTGGTCCTCATGGCTCAGCTTGTAAGTAACAAGAGGCGGAATTAGAGCAC
11	ALK19_11	AGATCCCCAGACACCAATTCAGAGATCTTTTCATGATGTGGCTCTTCTCCAACTCTGTGGCTTGGCAGTTCTCCAACTATAGGAAACACAACTGACCAAGATCCCAGCTGCACCCTCAAA
12	ALK19_12	TCCACTGCTGTGATTGCACTGAAGCTGCCCTACCCAATGGCTGAGCACAGCAGAAATACTAAGGCAGGCCAATTCCTGGGAGTCATGGGACTCCTCTGATGACTGACTTTGGCTCCAGAA
13	ALK19_13	CCCCTTAGGGCCTTGCTGAAACTTCCTTAGGCTCCATGGCACCCAGGGTGCTTCCACCCAACCTTCCCTCCCTCCCTCGTTCACGTGGGGTTATACTTGCAACACAGTCTGCTGGTTCAC
14	ALK19_14	CCAGCCTTCCCTGGCTCCCTCCCCATTTCCTCTCATGGGCATTTCTTCTAATAAAATCTGCAGACCATATTGGGTCTAATCCCATCTCCAGTCTGCTTCTTGGAGGAACCAGACTAACAT
15	ALK19_15	GACTCTGCCCTATATAATACAAATAATTATTTTCCATATATCTGATTTTTAGCTTTGCATTTACTTTAAATCATGCTTCAATTAAAGACACACCTTCTTTAATCATTTTATTAGTATTTC
16	ALK19_16	TAAGTATGATGGAAAGGTTCAGAGCTCAGGGGAGGATATGGAGATCCAGGGAGGCTTCCTGTAGGAAGTGGCCTGTGTAGTGCTTCAAGGGCCAGGCTGCCAGGCCATGTTGCAGCTGAC
17	ALK20_1	GGTGAGCGCCCTGCTGCCGTCCTGGGAGGAGAGGGGTGCAGTGTAGGGGCTGAATGTTATCACAGCACCGCAGACTCCTCTAGCCACAAAAGGCCGGCAGAGCCCTCCCTATGGGCACCC
18	ALK20_2	CTAGCCCTCCTAGGAGGACAAGCCTTGACATTCAGGGCCATGTATGTTGGCTTACATTAACTCCCATAGTTTATGGAGTTTATACAATGAGAGGAGAACCAGGGCTCTCCTCAAAATTCA
19	ALK20_3	TTCAGATGTGCTCTCTCCAAGTCTACCTGGCTCCCCTTCCCTCCAGGGGAGCAGGGGAGCAAAGGGCCCCTCCTCTGAGCATCTAGAGCAACTGCGCCTTCCTCTGTCACTCACTGGAAA
20	ALK20_4	TACTCCCCAGCCAGTACCCACAGTGATCTGTCTCTCCCCAAGGAGCATGAACCTCCCCCGGGGAAAAGCCATGGCTTATGCACCCCTGTGTCCCTGGCAAATATCAGAGACTCAATAACT
21	ALK20_5	ACTGATTGAATGAATAAACATTTGACCATTGTCATGAGTCCCTGGTTGGAATCCTTCTTACCAGTTTTCAGGTGAAGAACTGGAAGCCCGAGATTGCATAGCAAAGCCATGTTGAGGGTA
22	ALK20_6	TTACTCCTGAGTGTGTATGTTACCCCCGCCTCTCGTGTTTGTCCACTAAATGTGACGCCCAGGCTCAGGACCCCCAGCTGCCTCATTATTGTGGCCTGTTTGACTCTGTCTCCTCTTGTC
23	ALK21_1	GGTAAGAAGTGGCTCACTCTTGAGCCTGCCCTTGGCTTGCGGACTCTGTAGGCTGCAGTTCTCAGCTCACAGCCTCCTCCTCCTCCCCACCCTCCCCTTCTCTGCCCAG
24	ROS131_1	TGGAAATACAATGAGTTTTACCATGTTAAAACTTCATGCAGCCAAGGTCCTGCTTATGTCTGTAATATCACAAATCTACAACCTTATACTTCATATAATGTCAGAGTAGTGGTGGTTTAT

25	ROS131_2	AAGACGGGAGAAAATAGCACCTCACTTCCAGAAAGCTTTAAGACAAAAGGTGAGTACTAACATACTCAATACTCAGAAAACTCTGTGTTTCAGTTTTTTTCTTATCACTTATTTCACTAA
26	ROS131_3	TCCTAGAGATATATGAAATTGTTCTTTAAAAGCAGTACATTATTATTTCTAGAACTTGAATGAAGAAATTCAGAAAATATAGGAGACCTTGCAGGCAGTAATGAAATAATACTGGCTGGG
27	ROS131_4	TCTGGCTGGATCCTGAACTGGGCAAAATTACTTCTTCTTTCAGTATCCTTATTTCATGTCTAGCTATGACAATTTTATGGTGATAACTATATGAAAATAGTGTTACTGAATGTTCAAAAA
28	ROS131_5	ATATTTATTGGCTACTATATTTGAGATACTAGGATGAAAAATATAAGAAGTTCATTAAAAATTAACTAGTGTTCTGAAAGATATACCTTAACTCTGAGATGAGCTCTATAAGAATGGAAG
29	ROS131_6	GAACAAAGATCTAGAACCAAAAATACCATTTGACCTAGAAATTACATTACTAGTTACATACCCAAAGGAATATAAATCATTCTATTACAAAGATACATGCACACGTATGTTCACTGTAGC
30	ROS131_7	ACTATTCACAATAATGAATCATTTGGAATCAACCCAAATGCCCGTCAACATTAGACTGGTTGAAGCAAATGTGGTACATATACACCATGGCATACTATGCAGCCATAAAAAGGAATGAGA
31	ROS131_8	TAATGTTTTTTTGCAGGGATATGGATGAAGCTGGAAGCTATTAACCTCAGCAAACTAATTCAGGAACAGAAAACCAAACACTGCGTGTTCTCACTTACAAGTGGGAGCTGAACAATGAGA
32	ROS131_9	ACGCATGGACACAAGGAGGGGAACAACACACACTGGGGCCTGTCAGGGGGAGGGGTGGGGGGATAGAGAGTATTAGGAAAAATAGCTAATGCATGCTGGGCTTAATACCTAGGTGATGGT
33	ROS131_10	TTGCTAGGTGTAGTAAACCACCATGGGACATGTTTACCCATGTAACAAACCTTCACGTCCTGCACATGTACCCCAGAACTAAAAATAAAAAAATTTGAAAAAAAGAATGGAGGGAAAATG
34	ROS131_11	CTCCAAAACTTATGATAGTTTTCTTTATTTTCATTTATCACTTATTTTGTTTCAAGTTGAGATATATTGTTAATCTTATCACCAATTCATCAAATCCATCACTAATAAAAACTTCAAATG
35	ROS131_12	CTCATTTAAAAATATGTAATCATATAAGAATATTTCTGAATATTTCTTTTTTTAAAATTTCTTCTTAAAAAATAAGATACATGTGCAGAACGTGCAGGGTTGTTACATAGGTATACATGT
36	ROS131_13	GCCATGGTGGTTTGCTGCACCTATTGACCTGTCCTCTAAGTTCCCTCCCCTCAGCTCCTATCCCTCAACAGGCCCTGGTGTGTGCTGTTCCCCTCTCTGTATCCATGTGTTCTCAATGTT
37	ROS131_14	CAACTCCCACTTATGAGTGAGAACACGTGCTGTTTTGTTCTCTGTTCCTGTGTTAGGTTGCTGAGGATGATGACTTCCAGCTTCATCCATGTCCCTGCAAAGGACATGATCTCATTCCTT
38	ROS131_15	CTTATGGCTGCATAGTATTCCATGGTGTATATGTACCACATTTTCTTTATCTGGTTTGTCATTGATGGATTGGCTTGGTTCCATGTCTTTGTTATTGTAAACAGTGCTGCAGTAAACATA
39	ROS131_16	CATGTGCATGTGTCTTTATAGTAGAGTGATTTATACTCCTTTGGGTATGTACTCAATAATGAGATTACTGGGTCAAATGTTATTTCTGGTTCTAGATCCTTGAGGAATTGCCATACTGTC
40	ROS131_17	TTCCACAATGGTTGAAATAATTTACTTTACCACCAACAGTGTAAAAGTATTCCTATTTCTCCACACCCTCATCAGCATCTATTGTTTCCTGACTTTTTAATAGTTGCCATTCTGACTCGC
41	ROS131_18	GTGAGATGGTATCTCATTGTAGTTTTGATTTGTACTCCTCTGATGATCAGTGATGTTGAGCTTTTTTCATACGTTTATTGGCTGCATAAATCCCTTTTTGAGAAGTGTCTGTTCATATCT
42	ROS131_19	TTTGCCCACTTTTTGATGGGGTTGTCTGTCTTTTTCTTGTAAATATGTTTAAGTTCCTTGTACATTCTGGATATTAGCCCTTTGTCAGATGGATAGATTGCAAAATTTTTCTCCCATTCT
43	ROS131_20	GTAGGTTGTCTGTTCACTCTGATGATAGTTTCTTTTGCTGTACAGAAGCTCTTTAGTTTAGTTAGATCCCATTTGTCTATTTTGGCTTTTGTTGCCATTGCTTTTGGTGTTTTAGTCATG
44	ROS131_21	AAGTCTTTGCCCATGCCTATGTCCTGAATGGTATTGCCTAGGTTTTCTTCTAGGGTTTTTATGGTTTTGGTTTTTACATTTAAGTGTTTAATCCATCTTGAGTTAAATTTTGTATAAGGT
45	ROS131_22	TAAGGAATGGGCCCAGTTTCAGTTTTCTGCATATGGCTAGCCAGTTTTCCCAGCACCATTTACTAAATAGGAGATCCTTTCCTCATTGCTTGTTTTTGTTAGGTTTGTCAAAGATCAAAT
46	ROS131_23	GGTTGTAGATGTGTGGTGTAATTTCTGAGGTCTCTGTTCTGCTCCATTGGTCTATATATTTGTTTTGGTACCAGTACCATGCTGTGTTACTGTAGCTTTGTAATATAGTTTGAAGTCAGG
47	ROS131_24	TAGCATGATGCCTCCAAGTTTGTTCTTTTTGCTTAGGATTGTCTTGGCTACATGGGGTCTTCTTTGATTCTATGTGAAATTTAAAATAACTTTTTCTAATTCTATGAAGAATGTCAACGG
48	ROS131_25	TAGTTAGATGAGAATAGCATTGAATCTATAAATTACTCTGGGCAGAATGGCCATTTTCATGATACTCATTATTCCTGTCCATGGGGATGGAATGTTTTTCCATTTATTTGTGTCCTTTCT
49	ROS131_26	TATTTCCTTGAGCAATGGTTTGTAGTTTTCCTTGAAGAGGTCCTTCACATCCCTTGTTAGCTGTATTCGTAGGTATTTTATTCTCTTTGTAGCTATTGTGAATGGGAGTTCATTCATGAT
50	ROS131_27	TTGGCTCTCTGCTTGCCTATTGTTGGTGTAAAGGAATGCTTGTGATTTTTGCACATTGATTTTTTATCTTGAGACTTTGCTGAAGTTGCTTATCAGTTCAAGAAGTTTTTGGGCTGAGAT

51	ROS131_28	GATGGGGTTTTCTAAATACAAAATCATGTCATCTGCAAACAGAGACAACTTGACTTCCTCTCTTCCTATTTGAATACCCTTTATTTCTTCCTCTTGCCTGATTGCCCTGACCAGAACTTC
52	ROS131_29	CAATACTATGTTGAATAGGAGTGGTGAGAGTGGACACCCTTGTCTTGTACTGGTTTTCAAAGGGAATTCTTCCAGGTTTTGCTCATTCAATATGATATTGGCTGTAGGTTTGTCATAAAT
53	ROS131_30	AGTTCTTATTATTTAGAGATATGTTCCATAAAAACCTAGTTTATTGAGAGTTTTTAACATAAAGGGATGTTGATGTTGAATTTTATCAAAGGCCTTTTCTGCATCTATTCAGATAATCGT
54	ROS131_31	GTGATTTTTGTCTTTGGTTCTGTTTATGTGATGGATTACATTTATTGATTTGCATATGTTGAATCAGCCTTGCATCCCAGGGATGAAGCCGACTTGTTCGTGGTGGATATGTTTTTTGAT
55	ROS131_32	GTGCTGCTGGATTCAGTTTGCCAGTATTTTATTGAGGATTTTTACATCAATGTTCATCAGGTATATTGGCCCGAAGTTTGTTGTTGTGTCTCTTCCCAGTTTTGGTATCAGGATGATGCT
56	ROS131_33	GGCTTCATAAAATGAGTTAGGAAGGAATTCCTCCTTTTCCATTGTTTGGAACAGTTTCAGAAGGAATGGTACCAGCTCCTCTTTGTATTTCTGGTAGAATTCAGCTGTGAATCCATCTGG
57	ROS131_34	TCCTGGGCTTTTTTTGGTTGGTAGTCTATTAATTACTGCCTCCATTCCAGAGCTTGCTACTGGTCTAGTCAGGGATTCAACTTCTTCCTGGTTTAGTCTTGGTGTATGCATCCAGGAATG
58	ROS131_35	TATCCATTTCTTCTAGATTTTCTAGTTTATTTGCATAGAAGAGTTTATAGTATTATCTGGTGGTATTTTGTATTTCTGTGGGGTCAGTGGTGATATCCCCTTTATCATTTTTTTTGTGTC
59	ROS131_36	TATTTGATTCTTCTCTGTCTCCTTCTTTATTTCTCTAGCTAGTGGTATATTTTGTTATTTATTTTATTTTTTAAAAAAAACGGCTCCTGGATGTGTTGATTTTTTTTGGAGGGCTTTTTG
60	ROS131_37	TGTCTTTGTCTCCTTCAGTTCTTCTCTGATCTTAGTTATTTCTTGTCTTCTGCCAGCTTTTGGATTAGTTTGCTCTTGCCTCTCTAGCTCTTTTAATTGTGATGTTAGGGTGACGATTTG
61	ROS131_38	AGATCTTTCTGGCTTTCTAATGTGGGCATTTAGTGCTATGAATTTTCCTCTTAACACTGCTTTAGTTGTGTCCCAGAGATTCTGGTACATTGTCTCTGTTCTCATTTGTTTCAAAGAACT
62	ROS131_39	TATTTATTTCTGCCTTAATTTCATTATTTACCCAGGAGCCATTCAGGAGCAAGTTGTTCAATTTCCAGGAAATTGTGTGGTTTTGAGTGAGTTTCTTAATCCTGGGTTCTAATTTGATTG
63	ROS131_40	CTCTGTGGTCTGAGAGACTGTTTGTTATGATTTCAGCTCTTTTGCATTTGCTGAGGAGTGTTTTACTTCCAATTATGTGGTAGATTTTAGAATAAGTACCATGTGGCACTCAGAAGAATG
64	ROS131_41	TATATTCTGTTGATTTGGGCTAGAAAGTTCTGTAGACATCTACTAGGTCCACTTGATCCAGAGCTAAGTTCAAGTCCTGAATATCCTTGTTAATTTTCTGTCTTGTTGATCTGTCTAATA
65	ROS131_42	CTGGCAGTGGGGTGTTGAAGTTTCCCACTACTATTGCGTGGCAGTCTAAGTCTCTTTGTAGGTCTCTAAGAACTTACTTTGTGAGTCTGGGTGCTCTTGTATTGGGTGCATATATATTCA
66	ROS131_43	GAATAGTTAACTCTTCTTGTTGAATTGTTCCCTTTACCATTATATAATGCCCTTCTTTGTCTATTTTGATCTTTGTTGGTTTAAAGTCTGTTTTGTCAGAGACTAGGATTGCAACCCTCT
67	ROS131_44	TTTTTTTTTTTTTTGCTTTCCATATGCTTGGTAAATTTTCCTCAATCCCTTTATTTTGAGCCTGTGTGTGTCTTTGCATGTAAGATAGATCTCCTGAATACAACACACCAATGGGTCTTG
68	ROS131_45	ACTCCTTATCCAATTTGCCAGTCTGTATCTTTTAATTGGGGCATTTAGCCCCTTTACATTTAAGGTAGGTATTGTTATGTGAGTTTGATCCTGTCACCATGATGCTATTTGGTTATTTTG
69	ROS131_46	CATGCTAGTTGATGCAGTTTCTTCATAGTGTCATTGATCTTTATAGTTTGGTGTGTTTTTGCAATGGCTGGTACCAGTTTTTCCTTTCCATATTTAGTGCTTATTTCAGGAGCTGTTGCA
70	ROS131_47	GGGCAGGCCTGGTGGTAACAAAATCCCTCAGCATTTGCTTGTCTGGAAAGGATTTTATTTCTCCTTCACTTATGAAGCTTAGTTTGGCTGGATATGAAATTCTGGGTTGAAAATTCTTTT
71	ROS131_48	CTTTAAGAATGTTGATGTTGGCCCCCAATCTCTTCTGGTTTGTAGGGTTTATGTTGAGAGGTCCACTGTTAGTCTGATGGGCGTCCCTTTGTAGGTAACCTGGCCTTTCTCTCTGGCTGC
72	ROS131_49	CCTTAACAGTTTTTACTTGATTTCAACCTTGGAGAATCTGATGATTATGTGTCTTGGGGTTGATCTTCTCGTGGAGTATCTTAATGGTGTTCTCTGTGTTTGCCGAATTTGCATGTTGGC
73	ROS131_50	CTGTCTTGCTAGGTTGGGGAAGTTCTCCTGGATAATATCCCGAAGGGTGTTTTCCCACGTGTTTCCATTTTCCCCATCTCCTCCTGGTACTCCAAGCTATCATAGGTTCAGTCTTTTTAT
74	ROS131_51	GAAGTCTCATATTTCTTGGAGGCTTTATTCATTCCTTTTCATTCTTTTTTCTCTCTTCTTGTCAGCATGTCTTATTTCAATAAGGTAGTCTTCAAACTCTGATATCTTCTCTTCCACTTG
75	ROS131_52	GTCGATTCAGCTGTTGACACTTGTGTATGCTTCACGAAGTTCTTGTGCTGTGTTTTTCAGCCCCATCAGGCCATTTGTGTTCCTCTCTAAACTGATTATTCTAGTTAGCAATTCCTCCAA
76	ROS131_53	CTTTTTATCAAGGTTCTTAGCTTCTTCGCATTGGGTTAGAACATACTCCTTTAGCTCATCATAGTTTTTTCTACCCATCTTGTGAAGCCTACTTCTGTCAGTTCATCCATCTGATCCTCC

77	ROS131_54	GCCCAGTTCTGCACCCTTAATGGAGACATTGTGATCATTTAGAGGAGAAGAGGCACTCTGGCCTTTTGGGTTTTCAGCATTTTTTTCATTGGTTCTTTCTCACCTTCGTGAGTTTGTCTA
78	ROS131_55	GTTGTGGTCTTTGAGGCTGCTGACCCTTGTATGGGTTTTTTGTGGGGGCCCTTGTTGTTGTTGTTGATGATGATGCTGTTGTTGTCACTTTCTGCTTATTTGTTTTTCTTTCAATAGTCA
79	ROS131_56	CTTCCCTCTTCTGTAGGGCTGCTGCAGTTTGCTGGGGTTCACTTTAGGCCTTATTCATCTGATTTGCTCCCACGCCTGGATATGTAACTCAAGGAGGCTGGAGAGCAGCAAAAATGGGTG
80	ROS131_57	TCTGCTCCTTCTTCTGGGACCTCTGACTTCAAGGGGCACCAACCTGATGCCAGTAGGATCGCTCCTGTATAGGGTGTCCTGACAATCCCTGTTGGAGGGTCTCACCCAGTTGGGTGGCAC
81	ROS131_58	CAGGAGGAGGACCCATTTAACGAAGCACTTTGTCCCCTGGTGGAGAGGGTGTGTTTTGCTGTGGGAAAGCCCACTTGTCTGGGCTTCCCAGATTCCTCAGAACTACCAGGAGGAGAGGCT
82	ROS131_59	AAGTCTGTTGGTCCGCAGAGACTGCAGCCACCCCTCCCCCTAGGGGCTCAGGCCCAGGGAGATCTGAATTCTGTCCCTGAGCCTCTGGCTGCAGTTACTGAAGATTCTGCAGGGAAGCCC
83	ROS131_60	CACCCACTGAGGAAGGATGGGTGAGGGTTAGACCTGAAGAGGCACTCTGGCCGCTGACTACCACAGCTGGTGTGTTGGGTTGTGGCGACAAGTCTTGGGACCAAGCTGTCCAGCCTCGCT
84	ROS131_61	GGCTCCCGCAGGGGAAAAGCACAGCCTGGAGCTATAGAAATGGATGCTGCATTTCTCCCCGCCGCCCAGGGAGATTAGCGTGTTAGGCAGTTGGGAGTCCCAGTGCTGGCTGCTGCCCCT
85	ROS131_62	CCCTCAAGGAGCTCAAAGGGCTTAGACAGCAGGCAGCAGAAGCCTGTGCTGGTCACCCCTCCCCCAGGGAGTTCAGTAAGCTTAGGCAGATTCCAGCTGAGAGGCCATAAGAATCTGTAC
86	ROS131_63	ATTCTAGGGTTGGGATGCTAGGCCTCGGTGGCATGGGATCGAGAGTGGGATCTTCCAATCCATGAGTTGCACAGTTCTTTGGAAAAGCAATTTCCCCGGCTGGGTAGCCTGCTCGCTCGC
87	ROS131_64	CGCTCCCCTTGGCTGGAGGGGAGGGGGTTCCCCTTCCCCGTGTGGTTCTCAGGTGGGCTCACTGCTCTTCCTTCTCTCTGTGGGTCACGCCAGCCTTCTAGTCAATTTTGATGAGCGAAT
88	ROS131_65	CTAGATACCTTGCCGGTGAAGGACTCACACACTTATTATGGTTTTTTTCAATAGGAACCTCTGAACGGAGTTGCTTCTAGTCGGCCATCTTGGCCCCGCCTCTGAATATTTCTTTAATGT
89	ROS132_1	GGTATGTGTGTTTGCAAAGTACTTGTAAATGACAAAGAACTAAGAAGATAATTATAAAGTAATCAAAAGTAACACTATTTATGCAAATGTATTTATATACAAACACAAAGATCTTTAGTT
90	ROS132_2	AAAGGACTCACATGCATCATTACGTTCTTTGCTCAATTCCTAGACGTTGGTCTTGGCATGCTAATTATAAACAGATCACGTCATTCTCAGAAATCTCTAACTGCTCATTGACACTTTATG
91	ROS132_3	GAAATTGTTTGATCATAGGCTCATGCAACCAGTATTAACTTAATATCAGTTTTGTTTAAAAAGCTTAGCTGATGTTTAATGTTAAATAATGATGGCATGTAAATTCCTGATGATAATTTG
92	ROS132_4	CTTTAGCAAGGTGAATACTCTCTAGTGAGAAATGGTGATGAAGTAGATATTTCTCATACTGTAAAATTAGGTCAGGTTCTGAAATGGTCTGAAGTGTAGAGGTCACACAGGATTCTGTAT
93	ROS132_5	TCATTGGGAAGATTACAGTTTGCAATGTGTTCCCAGGGACTGAAAACACACCGTCGATGAAAACCAGCCACTGATGAACAGCCTCAGACCTGGCCAGTACGGGGAGCACGTGAACCATAA
94	ROS132_6	CTGGCTTAGAAGGACTGATTTCATGCCAACTTGAGGGGAGAATTCCAAATTCCAAAGACACTCCTCTCACAGGACTATGAAACCATCTTTTAAGAAGTTCCAAGGACAAAAGTCACTTCC
95	ROS132_7	ATAATTATAGTCCTGGCAACTTGATCTTTAGATCTATTTTTGCTTCATCTGGCTCTGGCATTCCCATAACCTTAAAAAATGTTCCTGTTCATACTAGAGAATTCCCTTGGCCCTTATTTC
96	ROS132_8	TAATTGAAACCTCCTTGAAGTTTCTGTTGCTGCTTTTCATGCAGAGTGAAATACATTCTAACCTGCAGAACTGTCTTGGTGGTTTGTGACTGTAATGATGTTCATCAATCAGGTCACACA
97	ROS132_9	TCAGGTTTTTGCTAGGACCTCTTTTGTGTGTTACCCTGAGTAACACCTTAAGTGCTACTAAAATGACTCCATCTAAATACTTTGCATACTCCTTGCTCCAGGTCTCCACTGTCAGGACAT
98	ROS132_10	AGACTATAGGATGACTGAATGATTGTTGTAACTTCAGTGTCTCAAAATAAGTCCTTCAGTCAGGACACTCTAGTTAGACTCTGTGTACTCACTGATGGGGTAGTGGAAGTGCAAAGTGCA
99	ROS132_11	TGAACTTGGGAGTTGAACAAGCCTTATTAGTTTCCAAGGGTTGCCACAATAAAGGACCACCAACTGGGTGGCTTGAAACAGTAGACATTTATTCTTACAGCTCTAGAGGTTAGAAGTCTG
100	ROS132_12	AAACCAACGTATCAGCGGGACTGCTTGTTCTGATGGTTCTAGGGCAGAATCCATCTTTGCCTCTTAGCTTCTGGCGTTTACTGGCAGTCCTTGGTATTCCTTGGCTCGTAGGTGCATCAC
101	ROS132_13	TGTCATCATCACCTCCTGCATCACAAGGTGTTCTCTGTGTCCAAATTTCCCTCTTCTTATGCAAGTGCCAGTCATTGGATTAGGGTCCACCCTAAACTACTATGACCTCATCTTAACTTG
102	ROS132_14	ATTACATTTGTCAAGATCTTATTTCTAAACAAGGTCACATTAAAATATTCTGAGTGGGTATGAATTTTGGGAGGACATGACTCAATCCAGTACACAAACCATGTTTAAATGCTTGTTCTT

103	ROS132_15	TTCCTTTTTTGAACTTAAGTTTCCTCATCTGTAAAATGGAGATGATAATATCTATCTTATCTCTTTAAGCTGTTGTGGACTCAGTGAATTAAAACAACGTTATGTAAAGCCCCAGTACAG
104	ROS132_16	TATGTTGTAAATAGAAGGTGCTCAACCAACATCAGAATTCTTCTTTCTTTTCTCAATGGCCATTGTGTAGAGCCACTTCACAAATTCTCAAGGGCCTCTTTCTTTTGGATCAGAGTTGTT
105	ROS132_17	TGGTTAAACAGTGAAATGGCCAATGCTAAAGGTGAGACGTCACAAAATGTGTAAAAACACTCTGTCCTAATGCTTTGCTCAGGATGGTCCTTAAAGGGACCCTCAGCTCCGGGGAACAGC
106	ROS132_18	TGTGACCAACTCTGCTGCCCTGCTGCTCCCGATGTTCTTACCCTTTCCCTGCCTCTTATGAGGGCCAGCCTTCACTCTCTGTTGGTCTTCAGAAGGATGAGTCAATTAGCAGGAATAGAA
107	ROS132_19	CCAGATTCTCCCACATATCCCTAAGCTTACCGTGGATTGGCACCAAGTCCTAAAACTATTGCGGTCTGAAAAGTTTCAGACCTGAAATATTCTTCATTGTTAGAGAAGACATAATGGAAG
108	ROS132_20	ACCTTGAGCTTGCCTTTGATGATTCTGTAAAAAGTCATTCCAGTTATGTCTTTTGAACCCAGCCCTTGTGCTCCTTCCATGTTTCAAATGAAAAACAATTGGATTGACTTTGAGGTGGAA
109	ROS132_21	TGTAGGTCAATACAAAAGGAAGCAACGAGCCTCAGAATCTCAGATATAATGATAACTTTGGTAGCTGAACTGTCACACTTTCCTTATCAATTTTGTATTCTGTATTTTATTGAAATATGG
110	ROS132_22	CATAAGTAAACTACAAATAATGTAAAAGACTGAAAATTGAGAAAATGGAAACTTCCCAGTTAAAAATCATGGAATAATTGTCTTATAGGTTTGGAATTCTGGAGAAAGTTTACCCATGCT
111	ROS132_23	GTTAGTATAGGAAAAAGGTACTCAGCTCTCTCTTCACCACTGATTACTCACAGTTTTCTTGGTGGGCTGAGTTAATTGGCTCCATGCCTCACTAGGATCACTATAAAACCATGCTGTTAG
112	ROS132_24	CATCTGTGTTGCCAGATTATGGGAGAGGAATGAGGTCAAGGGTAAATTGTGCCAAAGGACACTCTCTGGCGTTCAGTCACTAAGAAGATCTATGAACAACTAGAAATAACAGGTATATTG
113	ROS133_1	GGTATGTTACCATGTCTGTCTACACACTAGCTTATTACCTAAAGGTTCAGTAATAAATATCAGTACATTCTTAACATTAGCAATAGATAATGGTGACTATATATATATATATATATATAT
114	ROS133_2	ATATATATATATAATCTTATTATCCAAACTATTAAACTATTCCATTCATGTGATGTGATCTTTCTAAAACATCATTATCATCATGTCATTTTTCTCCCCAATAATAATCAGCCACCCCCT
115	ROS133_3	TAATTCCTACCACATCAATTCCATGCTCCTAATTAGGCTTCCAAGGCTGTTCACAATCTGTTCTATCCAGCCATATAATTCTATTCACATTATACCAGCCTTATGACCACTCCTACATTA
116	ROS133_4	TACATAACAGGCCTAAGACCTTTGTTTCTTCCCCAATATCTACCTCTTCCACTGTGTATTTTATTTTAGTTTATGATATATTGTCTTTCTAATCACATAGGCAGGAAATCTCAGTGCCAT
117	ROS133_5	ATGTGATTTTTCCCTCTACTCAGTAATCAGGTCCTGTTGATTTTACCATCTTGATTTTTCTCTGATGAGTTCTCATCTTCCTCTTTCCACCTTGGCTTTCCTAATTTAAGGTATCATTTC
118	ROS133_6	TTGCCTGTGCCCTTGACTTACGCATACTGCTGACAGTTAAATTTAGTTGAAGCACAGGCTGGATTACTTAATCCCTCTCTGAAATACCCACAATGGCTCTCCATTTACTGCTTTCAGAAT
119	ROS133_7	CAGCTACAAACTTCTTTGTGGCATGTGAGGTCTTCTGTAATTTATCCTCCAATTGTGGTTTATCTTTTCCAATATTTATGTCTGTGCTGTAGCCATATCAGACCAGTAAAAAGTTTTTAT
120	ROS133_8	GTCACTTAGTCTTTGGCCCTGTGTTTGCCTCAGCCTAGAATACAGGTCCCCACTACCTACCCTGTGCCCCTTAGCTGTGATTTCCTATTATTTATTTTCTGCCAATTGAAATTCTTCTCA
121	ROS133_9	TCCTTCAAGACTTAAATGAACTCATCATAATGCTTACCTGATGCTCCTTAGTCAAATGAATTATTGCATTTTATACACTCACATGCTATATAAAACTTACATGATTCTTGTTTATATTAT
122	ROS133_10	AGTTAGTTATCTAGTTAGTTGTGTACAGAAGTTTGCTACCCAGCTCAGCAAACTTTTTCTGAAAAGAACCAAATAGTAAATATGTTAGACTTTGCAGGCTACATGTGATCTCTGTAGCAT
123	ROS133_11	GTTCTTCTTTTCTTCTTTTTCTGTTCTCTTTTTCCTTCTCCTTCTTTTTAAAAATAACACTTTACAAAGGTAAAACTTATTCATAGCTAATAGGGGGTACAAAATCAGGCTATAGCCAGA
124	ROS133_12	TTTGGTCCTCATGCCATAGTTTGCCAGACTCTGGCCTACGTGTTTGTTTCCTCTACACAACTGAAACTACCTAAGAGAAATTACCATGTTTATTCCTCAGTTTAATATCCATGAAATTAA
125	ROS133_13	ATATGTATGAAGATATTATACAAAATAATAATGCCAACTATTTAGTATCCAAAGACTGAGATTTCTTGGTCCTAAATTTATTAAAAAGATATATATGTTTCCTAAGTCATTTTAAAGTAG
126	ROS133_14	AAGATTGAGTGGATATATTCCAGTGGTTTGTTGCTCTCTGCAAAAAAAAGCAAAAACACCTTGCTTTTGATTTCACATGGCATAAACACTGTCTGTATGGATGCTTTCAAGCTACCAACG
127	ROS133_15	GTCTAACAACTGGCTTGCAAAAATCCAGTAGTAGCTAGCTCTGCTATATTACTCTGTGTGCTTAGGTAGAGCTGGGGCAACTTAGCTTTTATCTATGAATTAATTTCTTTTTCTGATTTA
